# Systems-biology analysis of rheumatoid arthritis fibroblast-like synoviocytes implicates cell line-specific transcription factor function

**DOI:** 10.1038/s41467-022-33785-w

**Published:** 2022-10-20

**Authors:** Richard I. Ainsworth, Deepa Hammaker, Gyrid Nygaard, Cecilia Ansalone, Camilla Machado, Kai Zhang, Lina Zheng, Lucy Carrillo, Andre Wildberg, Amanda Kuhs, Mattias N. D. Svensson, David L. Boyle, Gary S. Firestein, Wei Wang

**Affiliations:** 1 Department of Chemistry and Biochemistry, University of California, San Diego, La Jolla, CA USA; 2 Department of Cellular and Molecular Medicine, University of California, San Diego, La Jolla, CA USA; 3 Department of Medicine, University of California, San Diego, La Jolla, CA USA; 4 Bioinformatics and Systems Biology Program, University of California, San Diego, La Jolla, CA USA; 5 Present Address: Division of Psychiatry, Haukeland University Hospital, Bergen, Norway; 6 Present Address: Department of Rheumatology and Inflammation Research, Sahlgrenska Academy, Institute of Medicine, University of Gothenburg, Gothenburg, Sweden

**Keywords:** Mechanisms of disease, Gene regulatory networks, Rheumatoid arthritis

## Abstract

Rheumatoid arthritis (RA) is an immune-mediated disease affecting diarthrodial joints that remains an unmet medical need despite improved therapy. This limitation likely reflects the diversity of pathogenic pathways in RA, with individual patients demonstrating variable responses to targeted therapies. Better understanding of RA pathogenesis would be aided by a more complete characterization of the disease. To tackle this challenge, we develop and apply a systems biology approach to identify important transcription factors (TFs) in individual RA fibroblast-like synoviocyte (FLS) cell lines by integrating transcriptomic and epigenomic information. Based on the relative importance of the identified TFs, we stratify the RA FLS cell lines into two subtypes with distinct phenotypes and predicted active pathways. We biologically validate these predictions for the top subtype-specific TF RARα and demonstrate differential regulation of TGFβ signaling in the two subtypes. This study characterizes clusters of RA cell lines with distinctive TF biology by integrating transcriptomic and epigenomic data, which could pave the way towards a greater understanding of disease heterogeneity.

## Abstract (web summary)

Fibroblast-like synoviocytes (FLS) are used as a model of rheumatoid arthritis synoviocytes, although cell lines derived from individual patients can have heterogeneous biology. Here the authors use a Taiji computational approach to analyze gene expression, chromatin accessibility and functional differences between individual patient-derived RA FLS lines.

## Introduction

Rheumatoid arthritis (RA) is a systemic inflammatory disease that causes joint inflammation and destruction^1^. A variety of targeted therapies, such as TNF blockers, have improved outcomes for RA patients, but a significant percentage have persistent inflammation. Treatment typically follows a trial and error method until a drug that decreases disease activity is identified^2^. Variable responses to therapy from patient to patient indicates diverse mechanisms of the disease, despite similar clinical phenotypes in RA.

There is considerable evidence of synovial tissue heterogeneity in RA^3^. A variety of approaches have used diverse technologies to predict therapeutic response, including histology^4^, immunostaining for cytokines like TNF, and composite scoring systems^5^. None of these, to date, have been successfully translated to clinical practice, although a recent biopsy study suggests possible correlations between the transcriptome and response to a targeted therapy^6^. Wide variability in synovial histology and infiltration by various innate and adaptive cell lineages in the joint further suggests that the important individual cell types can vary from patient to patient.

Fibroblast-like synoviocytes (FLS) form the synovial lining and have a major function in joint destruction in RA. Cultured FLS derived from rheumatoid synovium are epigenetically imprinted in RA and exhibit an aggressive phenotype with aberrant signaling^7^ that contributes to disease pathogenesis. Mesenchymal cell precursors in the peripheral blood of patients with RA have been associated with disease flares. We recently mapped the epigenomic landscape of cultured primary RA FLS, which demonstrated inter-patient heterogeneity but a stable repertoire of imprinted marks that distinguish RA from controls^8^. These differentially modified epigenetic regions identify diverse pathogenic pathways and potential therapeutic targets involved in immunity as well as unexpected processes such as the Huntington Disease Pathway.

We now report the application of the systems biology method Taiji^9^ to perform individual analysis on primary human cells. Our central hypothesis was that this algorithm could be applied to individual samples and that individual TFs can have different functions in primary cells derived from RA patients. This algorithm unexpectedly enabled clustering of FLS lines derived from individual RA patients into at least two groups that predicted context-specific TF function. Each cluster has distinct TFs that are predicted to have opposite effects on FLS function. Biologic validation experiments confirmed this hypothesis for the highly ranked retinoic acid receptor alpha (RARα) and demonstrated that it regulates distinct functions of the TGFß pathway in the two clusters. This dichotomy could explain the diverse effects of retinoids in RA animal models and why this has limited their development as a target for RA^10–13^. Our method provides a possible mechanistic explanation for the diversity of clinical responses to targeted agents in diseases like RA.

## Results

### Computational and experimental overview

To identify key driver TFs in rheumatoid arthritis we have applied Taiji to integrate genomic information from individual RA FLS lines into transcriptional regulatory networks. TFs were then ranked on their regulatory influence and cell lines clustered according to driver TF profiles. Computational assessment of the differences between these cell line clusters was conducted to identify important signaling pathways and define phenotypes. To validate these predictions, the following experiments were performed for the top ranked differential TF: ChIP-qPCR was conducted on cell lines from each cluster to assess predicted differential binding followed by siRNA knockdown of the top TF and RT-qPCR to assess downstream effects on gene expression, Western blot to assess downstream effects on protein, and MTT proliferation and Matrigel invasion assays to assess functional effects (Supplementary Fig. 1 and Supplementary Table 1).

### Global transcriptional gene regulation networks in rheumatoid arthritis

**Fig. 1:**
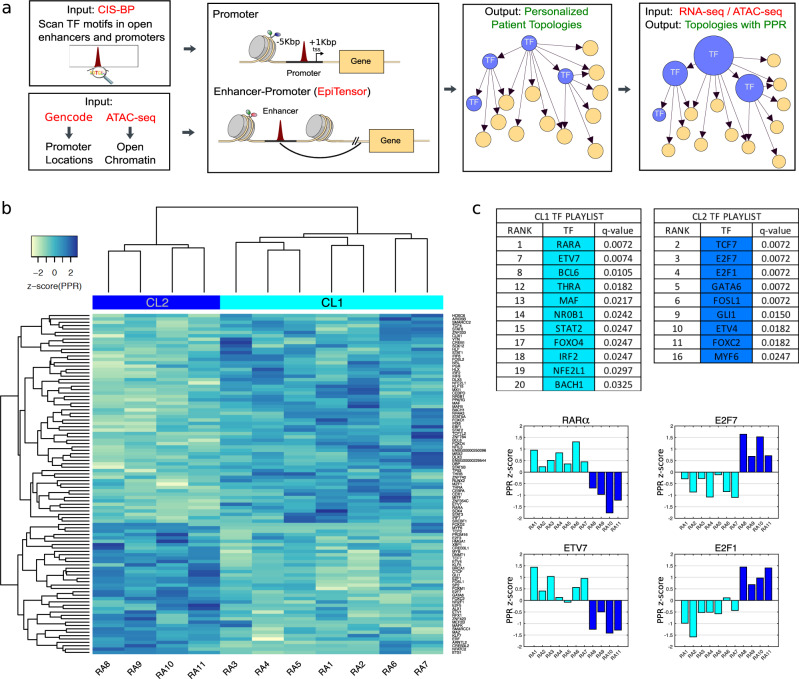
Individual transcriptional gene regulation networks for Rheumatoid Arthritis Fibroblast-like Synoviocytes allow for cell line stratification based on global TF regulatory differences. **a** Construction of cell line specific global transcriptional gene regulation networks using the Taiji Integrative Pipeline with RNA-seq and ATAC-seq data. Ranking of TFs using Personalized PageRank (PPR). **b** Cell line stratification into two clusters (CL1 and CL2) based on PPR. Hierarchical clustering using z-score(PPR) of top 100 TFs. **c** Top ranked TFs from a statistical power analysis of their regulatees and absolute change in PPR between CL1 and CL2. CL1- and CL2-specific TF “playlists”. Barplots of z-score(PPR) for top ranked cluster-specific TFs. Source data are provided as a Source Data file.

We constructed genetic networks in FLS from RA patients using the Taiji pipeline (Fig. 1a, “Methods” and Supplementary Fig. 2). Taiji first identified active regulatory elements using ATAC-seq peaks and then scanned the known 745 TF motifs documented in the CIS-BP database in these regions, to predict putative TF binding sites. The TFs whose binding sites were found in a gene’s promoter were linked to that gene (a topological “edge” between two “nodes”) else they were labeled as an enhancer site and linked to a gene using EpiTensor predictions. These regulatory interactions were assembled into a genetic network. TFs were subsequently ranked based on their global regulatory influence in the genetic network using the Personalized PageRank (PPR) algorithm. Each node was weighted according to its expression. The weight of each edge was determined based on the expression level of the parent node TF and strength of the TF-gene association as indicated by the ATAC-seq peak intensity and the motif strength. Using the node and edge weights, the Personalized PageRank algorithm iteratively determined the global importance of each node in the network. TF nodes that form a relatively large number of edges as parents to highly important child TF/gene nodes (associated with the expression of the child nodes) received higher ranks. Note that these TFs are not necessarily highly expressed themselves. In our previous studies, the Taiji pipeline identified key TFs that were validated using simulated data and experiments^9,14,15^.

### Stratifying RA cell lines using the TF PageRank scores

A unique feature of Taiji is that it can be applied to individual primary cell lines derived from RA patients. We built the genetic network in each RA cell line, based on which the PageRank score for each TF in each cell line was calculated. Using the top 350 TFs ranked by variance that were expressed in at least one cell line, the 11 cell lines from RA patients clustered into two groups using hierarchical clustering with the Euclidean distance measure and the complete agglomeration method, named cluster 1 (CL1, 7 cell lines) and cluster 2 (CL2, 4 cell lines) (Fig. 1b). For comparison, we analyzed similar data from 11 osteoarthritis (OA) cell lines through the Taiji pipeline. When individual OA FLS lines were clustered with the same method we again observed two clusters of OA cell lines. Distinct differential TFs were noted, as compared to RA, with a very small overlap (55 unique to OA and only 7 in common with RA) (Supplementary Fig. 3). This was not surprising because RA and OA have distinct disease mechanisms with functional enrichment analysis of the OA cluster-specific TFs resulting in many developmental pathways including “Activation of HoX genes during differentiation*”* (*p*-value = 2.47 × 10^−10^ from a hypergeometric test for over-representation).

We next identified the cluster-specific TFs for RA FLS clusters. TFs were ranked based on the absolute difference between the mean TF PageRank *z*-scores from CL1 and CL2. Among the top 200 TFs, we selected the top 100 TFs whose “regulatees” (child nodes) also showed significantly different PageRank *z*-scores between the two clusters (*p*-value < 5.0 × 10^−2^ using Hotelling T-Squared test, see “Methods”). From this list, we obtained 28 TFs (Top 20 shown in Fig. 1c) with a *q*-value < 5.0 × 10^−2^ (Supplementary Data 1). These cluster-specific TFs, such as the CL1-specific RARα, ETV7, BCL6, BACH1, NFE2L1, THRA, and NR0B1, and the CL2-specific TCF7, E2F7, E2F1, FOSL1, and FOXC2, collectively regulate the differential functions of the two groups of RA cell lines.

### Transcriptional network validation

**Fig. 2:**
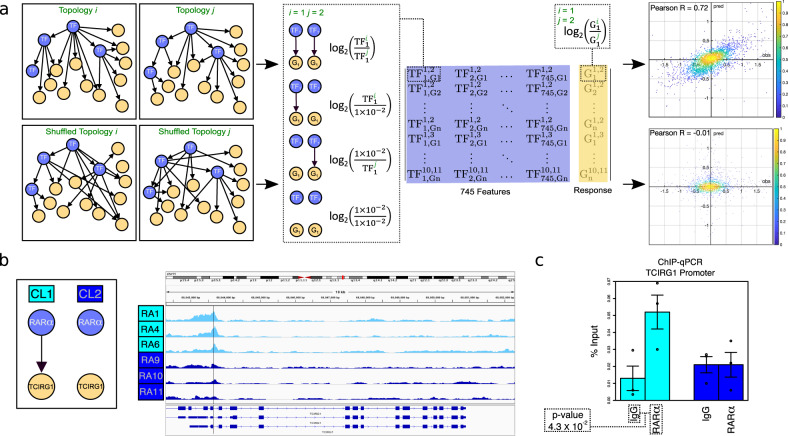
Computational and experimental verification of personalized transcriptional gene regulation networks for Rheumatoid Arthritis Fibroblast-like Synoviocytes. **a** Computational validation of predicted regulatory interactions using a 10-fold cross validated Random Forest regression model. **b** Selected differential edge formed by rank 1 cluster-specific TF, RARα →TCIRG1. ATAC-seq track from top 3 (CL1) and bottom 3 (CL2) patients as ranked by RARα PPR, showing TCIRG1 gene and location of ATAC-seq peak centered on RARα motif at chr11:68,043,640-68,043,657 (GRCh38/hg38) in the promoter of TCIRG1. **c** Experimental validation of cluster-specific predicted regulatory interaction using ChIP-qPCR showing CL1-specific RARα DNA-binding. p-value calculated by paired two-tailed Student’s t-test. Center line is mean and error bars +/− 0.5 standard deviation. CL1 *n* = 3 and CL2 *n* = 3 biologically independent cell lines. Source data are provided as a Source Data file.

Using the network topologies predicted by Taiji, the ratio of gene expression between all pairs of topologies (response) was predicted based on the ratio of expression levels of their specific parent TFs (features). We then validated the network by confirming the predicted gene expression differences. A Pearson correlation of 0.72 on the test sets using a 10-fold cross validation (Fig. 2a) was significantly higher than a value of −0.01 for randomly shuffled network topologies, which indicates that the predicted network topology captured important regulatory interactions.

We then confirmed the computationally predicted differential binding of the top CL1-specific TF, RARα, at chr11:68,043,640–68,043,657 (GRCh38/hg38) in the promoter of *TCIRG1*, a gene that has a function in bone remodeling and cancer metastasis^16^. Taiji predicted a regulatory interaction between RARα and *TCIRG1* in CL1 but not in CL2 (Fig. 2b). ChIP-qPCR on FLS lines was performed with the highest ranked (CL1) and lowest ranked (CL2) cell lines by RARα PPR score. CL1 lines exhibited a 4.11 mean fold change in yield (%input) compared to control (*p*-value < 0.05 by paired two-tailed Student’s t-test) whereas CL2 lines exhibited no change (Fig. 2c).

### The top ranked cluster-specific TFs regulate cell cycle and proliferation pathways

**Fig. 3:**
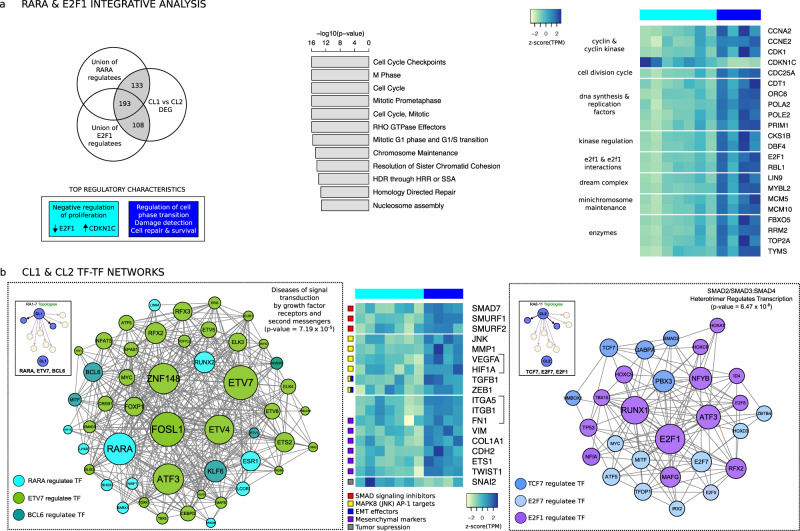
Network analysis for top cluster-specific TFs shows differential regulation of cell proliferation mechanisms and an imprinted TGFβ-driven EMT signature in CL2. **a** Union of RARα and E2F1 regulatees across all RA patient transcriptional networks intersected with CL1 vs CL2 2-fold (*p* < 0.05 by two-sided Student t-test) differentially expressed genes (DEGs). Functional enrichment analysis of high edge weight TF-specific DEG regulatees and RARα/E2F1 union DEG regulatees. Heatmap of gene expression (z-score(TPM)) for genes from selected pathway “*Mitotic G1 phase and G1/S transition*”. **b** Representation of CL1 and CL2 TF-TF subnetworks each using TF regulatees of the top 3 cluster-specific TFs with a significantly higher (*p* < 0.05 by two-sided Student t-test) edge weight in the given cluster. Edges between all network nodes shown and node size set to be proportional to out-degree. Nodes colored according to parent TF node identity (see legend). Functional enrichment analysis for all TFs in each network. Heatmap of gene expression (z-score(TPM)) for genes with significant differential expression (*p* < 0.05 by two-sided Student t-test) between clusters, related to Epithelial-to-Mesenchymal Transition (EMT), canonical SMAD-dependent TGFβ signaling and non-canonical MAPK signaling targets.

We investigated the patterns of pathways and regulatory function of top cluster-specific TFs, RARα, a cell cycle inhibitor, and E2F1, a cell cycle activator. We collated each regulatee for each cell line and then compared them across all RA FLS networks to define patterns of regulatees for each TF. Strikingly, the top CL1-specific RARα and CL2-specific E2F1 together regulate 74% (434/589) of the >2-fold DEGs, showing their importance in differentiating the two clusters. In particular, they regulate cell cycle pathways including cell cycle checkpoints, homology directed repair, G0/G1 and mitotic phases. Cell cycle activators are broadly overexpressed in CL2 and repressed in CL1, such as the CL2 overexpressed cyclin-dependent kinase CDK1 that drives cells through mitosis (Fig. 3a). RARα-specific pathways include “Negative regulation of mitotic cell cycle*”* (*p*-value = 1.53 × 10^−7^ from a hypergeometric test for over-representation) highlighting this. Notably, E2F1 regulates the tumor suppressor and inhibitor of cell cycle, *CDKN1C* (3.7-fold CL1, *p*-value = 2.7 × 10^−3^ by two-sided Student t-test)^17^.

These functions of the top cluster-specific TFs’ regulatees are consistent with those of all the 589 DEGs (expression change >2-fold and *p*-value < 5.0 × 10^−2^ by two-sided Student t-test) (Supplementary Data 2) between the two clusters. The most significantly enriched pathways include “Cell Cycle”, “Chromosome Maintenance”, “Cell Cycle Checkpoints” and “Signaling by Rho GTPases” (Supplementary Data 3). Taken together, these CL1 vs CL2 features point to differences in RA FLS cell cycle progression, adhesion and Rho GTPase signaling and cell cycle checkpoint initiation (Fig. 3a).

### TF-TF subnetworks predict differential regulatory functions of TGFβ in the two clusters

TF-TF subnetworks hold crucial transcriptional information. Therefore, we focused on the regulatees that are also TFs of the top ranked cluster-specific TFs (CL1-specific RARα, ETV7, and BCL6, and CL2-specific TCF7, E2F7, and E2F1, Fig. 3b). We identified the cluster-specific edges determined by the cluster-specific open chromatin pattern. We only considered the cluster-specific edges that were shared in >75% of the networks in the cluster that also had a higher edge weight (>1.5-fold) compared to the other cluster. We found that the CL1-specific TFs are enriched with the pathway “Diseases of signal transduction by growth factor receptors and second messengers” (*p*-value = 7.19 × 10^−5^ from a hypergeometric test for over-representation). In contrast, the CL2 TFs are involved in “SMAD2/SMAD3:SMAD4 heterotrimer regulates transcription” (*p*-value = 4.82 × 10^−6^ from a hypergeometric test for over-representation).

Analysis of transcriptomic signatures of repressors, such as *SMAD7* and *SMURF1,2* which are more highly expressed in CL2, supported the notion that the TGFβ SMAD-dependent signaling might be repressed but not eliminated in CL2 (Fig. 3b). Consistent with that concept, the angiogenic factors hypoxia-inducible factor 1-alpha (*HIF1A*) and vascular endothelial growth factor (*VEGFA*) are higher in CL2 with the latter being an AP1 target along with *MMP1* and *TGFβ1* (1.9-fold CL2, *p*-value = 1.6 × 10^−2^ by two-sided Student t-test). Non-canonical pathways such as the MKK7-JNK-AP1 axis represent TGFβ activated MAPK signaling casades^18^ that mediate RA FLS invasiveness^19^.

To explore other features that distinguish the two clusters of individual FLS lines, we examined histone modification differences using the integrative analysis pipeline EpiSig^8^ that identifies genomic regions with similar epigenomic profiles across 6 histone marks (H3K4me1/3, H3K27ac/me3, H3K36me3, H3K9me3). Ten clusters displayed high inter-patient variance in H3K4me1 signal and were enriched in the “SMAD binding” pathway (*p*-value = 1.79 × 10^−8^ from a hypergeometric test for over-representation) (Supplementary Fig. 4). This orthogonal analysis supported the Taiji result that integrated gene expression and open chromatin information.

### RARα differentially modulates downstream transcriptional responses in CL1 and CL2

**Fig. 4:**
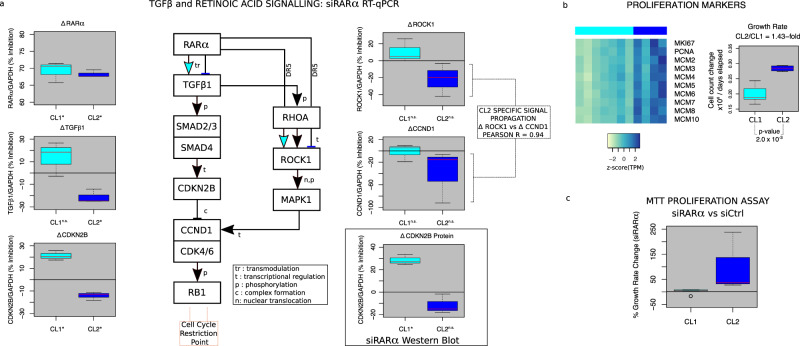
RARα divergently regulates TGFβ signaling and proliferation in a cluster-specific manner: transcriptional and functional validation. **a** siRARα RT-qPCR on CL1 and CL2 RA FLS. Boxplots of change in mRNA levels under RARα depletion (CL1: *p* = 0.041, CL2: *p* = 0.015) for TGFβ1 (CL2: *p* = 0.004) and canonical CDKN2B (CL1: *p* = 0.0009, CL2: *p* = 0.0002)/non-canonical signaling axis genes that undergo transcriptional regulation. Arrows (↑) illustrate activating and blunt ended lines (T) inhibiting/repressive effects. Cyan infill depicts the primary CL1 regulation logic, dark blue representing the primary CL2 regulation logic and black arrows representing both CL1 and CL2 logic. Boxplot of change in CDKN2B protein levels under RARα depletion (CL1: *p* = 0.048). *p*-values calculated by paired two-tailed Student’s t-test for siRARA vs CTL *p < 0.05. CL1 *n* = 3 and CL2 *n* = 3 biologically independent cell lines. **b** Heatmap of gene expression (z-score(TPM)) for genes with significant differential expression (*p* < 0.05 by two-tailed Student’s t-test) between clusters, related to cellular proliferation. Growth rate boxplot for CL1 and CL2 FLS. CL1 *n* = 5 and CL2 *n* = 4 biologically independent cell lines. **c** MTT proliferation assay boxplot for siRARα and siCtrl at 1 and 5 days after plating. CL1 *n* = 6 and CL2 *n* = 3 biologically independent cell lines. For all box plots, red center line for median, whiskers represent maximum and minimum values, box width from quartile 1 to quartile 3. Source data are provided as a Source Data file.

To validate the differential regulatory functions of TFs in the two clusters, we focused on a top CL1-specific TF, RARα, and investigated its differential effects on *TGFB* and its regulatees that were predicted based on the Taiji analysis. We therefore explored the possibility that the downstream TGFβ pathway has differential responses in CL1 and CL2 (Fig. 3b) by sequentially studying multiple steps down the TGFß pathway, anticipating a cluster-specific regulatory influence of RARα. We depleted RARα using siRNA, in the cell lines with the highest and lowest RARα PPR scores, followed by IL-1 stimulation. RT-qPCR showed that RARα mRNA levels were decreased by 67 and 68% in CL1 (*p*-value = 0.041 by paired two-tailed Student’s t-test) and CL2 (*p*-value = 0.015 by paired two-tailed Student’s t-test), respectively. This was confirmed at the protein level by Western Blot showing a decrease of at least 53 to 58% (*p*-value = 0.004 by paired two-tailed Student’s t-test) (Supplementary Fig. 5a). RARα deficiency had differential effects on TGFß1 expression in CL1 vs CL2 (18% decreasing trend in CL1 and a significant 25% increase in CL2 (*p*-value = 3.8 × 10^−3^ by paired two-tailed Student’s t-test)) (CL1 vs CL2 *p* < 0.05 by two-tailed Student’s t-test) (Fig. 4a).

We then confirmed that the pathway and genes downstream in the *TGFβ1* canonical signaling pathway, which initiates a series of SMAD-mediated steps, were differentially regulated in CL1 and CL2. The propagation of the upstream divergent effects were consistent through the pathway for CL1 and CL2. For example, we observed a significant 21% decrease in *CDKN2B* for CL1 (*p*-value = 9.21 × 10^−4^ by paired two-tailed Student’s t-test) compared with a significant 13% increase in RARα deficient CL2 cells (*p*-value = 2.43 × 10^−4^ by paired two-tailed Student’s t-test) (CL1 vs CL2 *p* < 0.05 by two-tailed Student’s t-test).

We also evaluated whether protein levels correlated with mRNA expression. These studies began with immunoassays of TGFß protein, recognizing that these measurements can be difficult to interpret because the growth factor binds avidly to matrix proteins^20^ and the functional effects require activation from the latent to the active form. We noted that latent TGFß1 protein levels were similar between CL1 and CL2 (Supplementary Fig. 5b). Because of issues with TGFß protein assays, we then evaluated a more tractable analyte by moving downstream to an intracellular TGFß regulatee, CDKN2B, which had similar differential mRNA expression in CL1 and CL2 (Fig. 4a). Using Western blot analysis, we confirmed that RARα deficiency had the predicted differential effect in CL1 and CL2 with a CL1 decrease in CDKN2B protein (*p*-value = 4.80 × 10^−2^ by paired two-tailed Student’s t-test) and no significant change for CL2 (CL1 vs CL2 *p* < 0.05 by two-tailed Student’s t-test) (Fig. 4a and Supplementary Fig. 5c). To evaluate if CDKN2B expression levels were due to aberrant SMAD signaling instead of decreased functional TGFß, we stimulated CL1 and CL2 FLS lines with TGFß and measured P-SMAD (Supplementary Fig. 5d). SMAD activation was similar in the two clusters, suggesting that differential CDKN2B expression is likely due to decreased TGFß in the microenvironment.

### Differential TGFβ signaling and biologic effects

SMAD-independent TGFβ signaling mechanisms can also initiate non-canonical effects, including activation of the cell cycle. In light of the known non-canonical TGFβ-mediated activation of RhoA signaling^21^, we analyzed the effects of RARα siRNA on the non-canonical RHO-ROCK-MAPK1-CCND1 axis (Fig. 4a). We observed a small decrease of median *ROCK1* expression in CL1 but an increase in CL2 after RARα knockdown (CL1 vs CL2 *p* < 0.05 by Wilcoxon Rank Sum Test) (Fig. 4c). Following the signal further, we only saw a trend of a change of *CCND1* transcription in CL2 (1% increase CL1 vs 38% increase in CL2), which correlated with the change in ROCK1 transcription (Pearson R = 0.94). To confirm that these observations had functional relevance and predicted differential responses to RARα ligation, we stimulated cells with RARα ligand all-trans retinoic acid and observed cluster-specific effects on cell growth (Supplementary Fig. 6).

### Cluster growth rate differences

Based on the previous findings that key cluster-specific TF functions regulate genes in the cell cycle (Fig. 4b), we predicted and then confirmed that CL2 FLS have a higher growth rate than CL1 FLS (*p*-value = 4.2 × 10^−2^ by two-tailed Student’s t-test). Furthermore, after confirming RARα’s differential regulation of TGFβ regulatees, we evaluated proliferation of the same cell lines after RARα knockdown. Figure 4c shows that RARα deficiency increased growth of CL2 cells but resulted in minimal change for CL1 cells. As shown in Fig. 4a, knockdown increased expression of individual genes that inhibit (*CDKN2B*) or stimulate (*CCND1*) proliferation. Therefore, the mechanism cannot be ascribed to a single gene but rather the integration of RARA regulatees leading to a net increase in proliferation after RARα knockdown in CL2 (Fig. 4a). This effect of RARα deficiency on growth contrasts with RARα activation, where the RARα agonist ATRA increased proliferation of CL1 but not CL2 cells (Supplementary Fig. 6).

### Transcriptional validation of predicted RARα CL1 and CL2 differences regulating matrix proteins

**Fig. 5:**
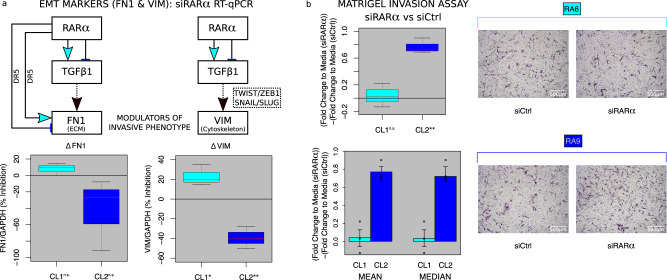
RARα regulates TGFβ signaling and Epithelial-to-Mesenchymal Transition (EMT) effectors in a cluster-specific fashion with subsequent transcriptional and functional validation. **a** siRARα RT-qPCR on CL1 and CL2 RA FLS. Arrows (↑) illustrate activating and blunt ended lines (T) inhibiting/repressive effects. Cyan infill depicts the primary CL1 regulation logic, dark blue representing the primary CL2 regulation logic and black arrows representing both CL1 and CL2 logic. Boxplots of change in mRNA levels under siRARα depletion for the EMT markers FN1 and VIM (CL1: *p* = 0.0189, CL2: *p* = 0.0041) (transcriptionally regulated downstream of TGFβ1). CL1 *n* = 3 and CL2 *n* = 3 biologically independent cell lines. *p*-values calculated by paired two-tailed Student’s t-test for siRARA vs CTL *p < 0.05 **p < 0.001. **b** TGFβ-stimulated Matrigel Invasion Assay boxplot for CL1 and CL2 (*p* = 0.0077) and barplot of mean and median values (error bars represent 1 standard deviation). *p*-values calculated by paired two-tailed Student’s t-test for siRARA vs CTL **p < 0.001. CL1 *n* = 3 and CL2 *n* = 3 biologically independent cell lines. Representative 4× magnification images of migrated RA FLS in siRARα and siCtrl for two exemplar patient cell lines RA6 (CL1) and RA9 (CL2). For all box plots, red center line for median, whiskers represent maximum and minimum values, box width from quartile 1 to quartile 3. Source data are provided as a Source Data file.

Alteration of the ECM (Supplementary Fig. 4) contributes to cell invasion. Analysis of the EpiSig clusters with significant CL1 vs CL2 differences identified two regions that are differentially marked by active enhancer marks H3K4me1 and H3K27ac as well as a strong H3K36me3 signal generally associated with transcription (Supplementary Fig. 7). Variable levels of these 3 marks in active enhancers may fine tune the expression levels of associated genes^22^. Of interest, these regions have significantly higher signal strengths for H3K4me1 (*p*-value = 8.22 × 10^−2^ by two-tailed Student’s t-test) and H3K36me3 (*p*-value = 6.59 × 10^−3^ by two-tailed Student’s t-test) in CL1. The related enriched pathways include “Degradation of extracellular matrix” (*p*-value = 1.65 × 10^−3^ from a hypergeometric test for over-representation), including fibronectin (*FN1*), and “Integrin cell surface interactions” (*p*-value = 6.29 × 10^−3^ from a hypergeometric test for over-representation). To assess a functional correlate of these observations, we evaluated the regulation matrix genes *FN1* and vimentin (*VIM*) in CL1 and CL2^23^. FN1 is transcriptionally regulated by TGFβ and RARα directly via DR5 RARE in the *FN1* promoter. RARα depletion in CL1 leads to a divergent trend toward inhibition of FN1 compared to a 27% increase of FN1 in CL2 (CL1 vs CL2 *p* < 0.05 by Wilcoxon Rank Sum Test) (Fig. 5a) in agreement with the predicted cluster-specific behavior of the DR5 motif architecture and downstream effects of TGFβ. After RARα depletion, *VIM* expression is decreased by 20% vs siRNA control in CL1 (*p*-value = 1.89 × 10^−2^ by paired two-tailed Student’s t-test) and increased by 40% vs siRNA control in CL2 (*p*-value = 4.10 × 10^−3^ by paired two-tailed Student’s t-test) (CL1 vs CL2 *p* < 0.05 by two-tailed Student’s t-test) (Fig. 5a).

### Functional validation of predicted effects of RARα on CL1 and CL2 invasion

Because the patterns of gene regulation were predicted to affect cell movement, we studied the effect of RARα on invasion through an artificial matrix. TGFß-stimulated FLS invasion after knockdown with siRARA was increased 77% compared to siRNA control in CL2 lines (*p*-value = 7.73 × 10^−3^ by paired two-tailed Student’s t-test) but there was no statistically significant change in CL1 lines (Fig. 5b).

## Discussion

RA treatment currently requires sequential use of therapeutic agents with distinct mechanisms until an effective combination is found^24^. The diversity of responses to these targeted drugs, such as cytokine blockers, T cell co-stimulation inhibition or B cell depletion, suggest that RA has a common clinical phenotype but multiple pathogenic pathways. There is currently limited understanding of these pathways and no way to translate them into therapeutic targets. Predictive biomarkers, such as synovial TNF expression or synovial histology assessments are not sufficiently predictive to be useful in clinical practice and do not provide information on disease mechanisms^4,25^. The present report suggests that context-specific TF function within gene and TF networks is unique for individual cell lines and could have implications for distinct pathogenic pathways in patients.

Fibroblast-like synoviocytes have a major function in joint destruction and inflammation in RA and assume an aggressive phenotype in the disease^26^. Increased numbers of mesenchymal elements known as PRIME cells presage disease flares in RA, supporting the concept that fibroblasts are important in this process^27^. Synovial fibroblasts can display a variety of phenotypes^28^, and cultured FLS have served as a useful tool to study disease pathogenesis. For example, an imprinted methylation pattern occurs in these cells^29^ and a comprehensive global characterization of the epigenomic landscape in RA FLS^8^ identified epigenomic dysregulation and pathogenic pathways. We now show that integration of epigenomic and transcriptomic data identifies marked cell line to cell line differences in pathogenesis and TF function in RA using a Taiji method that can be applied to any cell lineage or disease.

We developed an integrative systems biology method Taiji that integrates TF expression, binding and the expression of gene targets to construct gene-regulation networks for each cell line. The method further ranks TFs based on their relative importance in the network using the Personalized PageRank (PPR) algorithm. Taiji provides biological insights into mouse embryonic development^30^ and CD8+T cell differentiation^14^. We applied the algorithm to construct individual transcriptional gene-regulation networks for FLS from RA patients and study inter cell line heterogeneity. RA cell lines segregated into at least two groups with predicted distinct cell line-specific TF biology exhibiting divergent PPR scores between the groups.

Two groups, or clusters, were identified by Taiji, namely CL1 and CL2, that are characterized by the differential expression of cell cycle genes, cell proliferation, and EMT markers. Even with the relatively small number of patients in this study, the power of Taiji is its ability to leverage small differences between hundreds of genes (expression or open chromatin), subsequently integrating and quantifying them using complex transcriptional networks to identify the master TFs that regulate them. Hundreds of weak predictors are combined into dozens of strong predictors with the goal of identifying TFs with opposing regulatory functions via Taiji. Genes that encode TFs such as RARA, ETV7, E2F7 and E2F1 regulate cluster-specific differences in the core transcriptional machinery enriched in growth factor signaling (CL1) and SMAD-dependent TGFβ signaling TFs (CL2) highlighting differences in TGFβ growth factor signaling associated with a CL2 activated signature. Strikingly, we were able to identify TFs that were predicted to have a differential functional effects in CL1 and CL2.

We then biologically validated the predictions by highlighting the use-case TF RARA, a cluster-specific TF, whose regulatees show functional enrichment in growth factor signaling, mitotic cell cycle, cell cycle checkpoint, DNA repair, and also include EMT markers^31^. RARα, in conjunction with the heterodimeric partner RXRα, has ligand-specific transcriptional effects, and can act as a transcriptional activator or repressor dependent on the cellular context^32^. RARα knockdown in CL1 and CL2 cell lines confirmed the TFs predicted differential regulatory function with a decrease in TGFβ mRNA expression in CL1 and an increase in CL2. These effects were transcriptionally propagated downstream through SMAD-dependent signaling in both clusters and via SMAD-independent mechanisms in CL2. Functional assays showed that RARα depletion increases the proliferation and invasion of CL2 FLS but has little to no effect in CL1 FLS.

The biologic validation studies do not identify individual genes that are solely responsible for the differential effects of RARA. TF knockdown and activation engage the entire network and integrate the functions of many genes that can increase or decrease cell growth. Taken together, the data indicate that pro-proliferative effects of RARA deficiency in CL2 outweigh any anti-proliferative effects. It is the combination of all RARA regulatees, including CKDN2B (anti-proliferative) and cyclin D (pro-proliferative), that result in the internally consistent findings shown in Fig. 4c and Supplementary Fig. 6.

Dysregulation of the balance between FLS proliferative and apoptotic processes is critical to synovial lining hyperplasia and inflammation in RA. Subsets of RA patients exhibit increased synovial tissue inflammation and FLS from these tissues display gene profiles characteristic of myofibroblasts^28^. The use of Taiji builds of this concept of distinct pathogenic mechanisms, identifying a variety of TFs with differential functions like RARA, which interacts with TGFβ pathways that control proliferation and invasion.

Although the number of cell lines is relatively limited in the present analysis, the Taiji integrated systems approach compensates by increasing the statistical power through simultaneous assessments of hundreds of TFs and regulatees. One potential limitation is the focus on cultured RA FLS cell lines which are homogenous but do not necessarily reflect the diversity of fibroblast phenotypes in situ described by others^33^. However, cultured FLS and their pathogenic behavior are stable, can readily distinguish RA from non-RA, and have provided insights into disease mechanisms^34^. We were unable to analyze individual fibroblast phenotypes observed in situ using single cell technology but this methodology is rapidly advancing and could be feasible in follow on studies^35^. Furthermore, it would be important in future studies, to replicate these findings in a different cohort of RA FLS.

In conclusion, we present a method to stratify cell lines from individual patients with RA based on a global ranking of regulators in the genetic network constructed for each cell line. Hundreds of weak predictors are combined into dozens of strong predictors that can effectively characterize the distinct TF-patterns of individual cell lines and further provide a general tool for studying rheumatoid arthritis. The use-case of one TF, namely RARα, provides a foundation for the divergent response to retinoids in RA animal models which is highly variable including model-specific disease improvement or exacerbation.

## Methods

### Human FLS, RNA-seq and ATAC-seq

This study was approved by the Institutional Review Board of University of California, San Diego, and informed consent was obtained from all participants (protocol 14 - 0175). Synovial tissue was previously obtained from female patients (11 with RA and 11 with OA) at the time of total joint replacement or synovectomy with a prior two week discontinuation of any biologics, methotrexate, and leflunomide as previously described^34,36^. Cells were therefore not exposed to drugs for over 2 months prior to analysis. FLS were stained with Human TruStain FcX (Biolegend: cat#: 422302, lot: B290761) at 5 μL/million cells, then live cells were selected with ZombieRed diluted at 1 µL/million cells (Biolegend: cat#: 77475, lot: B308306) and detected with antibodies against CD34 (Biolegend: Alexa Fluor 647 anti-human CD14 - clone:63D3, cat#: 343610, lot: B301240), CD90 (Biolegend: PE- anti-human CD90 (Thy1) – clone: 5E10, cat#: 328110, lot: B301003), CD14 (Biolegend: Alexa Fluor 647 anti-human CD14 – clone:63D3, cat#: 367128, lot: B303259), Podoplanin (Biolegend: APC/Cyanine 7 anti-human Podoplanin – clone: NC-08, cat#: 337030, lot: B304084) and FAPa (R&D Systems: Alexa Fluor 647 anti-human FAP – clone: 427819, cat#: FAB3715R, lot: AFEM0319121), all at a concentration of 5 µL/million cells, on a ZE5 Cell Analyzer (BioRad). Analysis was performed with FlowJo, V10 (BD Biosciences) (Supplementary Table 2). Primary FLS lines were used due to the current limitations expanding recently identified synovial fibroblasts phenotypes that appear to display distinct functions and the current state of simultaneous determinations of chromatin accessibility and the transcriptome in single cells^33^. In the previous study^8^ cells were cultured in DMEM supplemented with 10% fetal calf serum and passaged 1:3 every one to two weeks. They were harvested for the epigenetic studies at the 5th passage when ~70% confluent. Genomic DNA from fibroblast-like synoviocytes (FLS) were isolated and total RNA was extracted with raw read quality evaluated using FastQC. Adapter and low quality bases were trimmed from the raw RNA-seq reads and further trimmed with reads less than 30 bp being discarded as previously described^8^. In this study, the reads from these data were aligned, assembled, and quantified by RSEM. ATAC-seq sample preparation followed the protocol found in Buenrostro et al.^37^ Peaks were called for each replicate experiment using the Irreproducibility Discovery Rate (IDR) framework to identify reproducible peaks. Peaks from individual replicates as well as pooled data from two replicates were called with MACS2 and a relaxed threshold (*p*-value = 1.0 × 10^−2^). The 3 sets of peaks were input for IDR analysis with a threshold of *p*-value = 5.0 × 10^−2^ for high confidence peaks as per Yu et al.^14^.

### Taiji pipeline 1: Network construction

Active regulatory elements were first identified via the overlap of high confidence peaks from ATAC-seq with known gene promoter regions (5 Kbp upstream and 1 kbp downstream of the transcription start sites), (Fig. 1a and Supplementary Fig. 2). The distal ATAC-seq peaks not assigned to promoters were then labeled as enhancer regions and linked with promoters using chromatin interactions predicted by EpiTensor. Putative TF binding motifs were curated from the CIS-BP database^38^. Using FIMO’s algorithm^39^, 745 TFs were identified as having binding sites within 150 bp regions centered around ATAC-seq peak summits. 11 unique network topologies were thus constructed by forming directed edges between TF and their regulatees, if the TF had a predicted binding site in the child gene’s promoter or linked enhancer.

### Taiji pipeline 2: Personalized PageRank (PPR)

The Personalized PageRank (PPR) algorithm was run to measure the global influence of each node (Fig. 1a and Supplementary Fig. 2). PPR is a link analysis algorithm that assigns a numerical weighting to each node in a network. Mathematically, PPR is the stationary distribution of a random walk. To initialize the networks, node weights were assigned according to the relative gene expression normalized across all 11 samples calculated as ${{{{{\rm{e}}}}}}^{{z}_{i}}$, where *z*_*i*_ is the *z*-score of the expression of the gene *i* in each cell line (expression quantified by RNA-seq). Edge weights were determined according to the expression level of the parent node TF and the pooled ATAC-seq peak intensity (strength of the TF-gene association) as outlined in Zhang et al.^9^. The normalized node weights were then used as the seed vector for the PPR calculation. Thus, if ***s*** is the vector containing node weights and ***W*** is the edge weight matrix then the PPR score vector ***v*** is calculated by solving a system of linear equations (Eq. 1)

1 $${{{{{\boldsymbol{v}}}}}}=\left(1-d\right){{{{{\boldsymbol{s}}}}}}+d{{{{{\boldsymbol{Wv}}}}}}$$

where *d* is the damping factor (*d* = 0.85 by default). Solving iteratively (Eq. 2) we have:

2 $${{{{{{\boldsymbol{v}}}}}}}_{t+1}=\left(1-d\right){{{{{\boldsymbol{s}}}}}}+d{{{{{\boldsymbol{W}}}}}}{{{{{{\boldsymbol{v}}}}}}}_{t}$$

### Network validation 1: Computational

Network topologies were validated according to the following protocol. The 56,269 transcripts, for which expression data were available, were ranked according to expression in each patient. For each of the 55 unique patient pair combinations of 11 patients, the unique union of the top 500 most highly expressed genes in each patient were obtained. For each gene, if an edge existed between the gene and a given TF for both patients in the given pair, the log_2_ of the fold change in expression values (transcripts per kilobase million (TPM)) was calculated (setting any 0 expression values to 1 × 10^−2^). If the TF to gene edge was not present for a given patient topology the expression value was set to 1 × 10^−2^ before taking the ratio and performing the log_2_ operation. Subsequently for each patient pair, each gene contained 745 features (for each pair of TFs), thus generating a 28,645 × 745 feature matrix. The response variable was calculated as the log_2_ of the fold change in expression for the gene (setting any 0 expression values to 1 × 10^−2^ as before). Using the feature matrix and response variable we performed 10-fold cross-validated random forest (RF) regression and subsequently calculated the average Pearson R correlation for the predicted values of each test set vs the actual response values. For randomized topologies, each row of features were randomly shuffled in the feature matrix, for the given response variable, and the same 10-fold cross validated RF regression performed.

### Network validation 2: ChIP-qPCR

Cells fixed in 1% formaldehyde were washed with PBS, scraped and pelleted. After cell lysis, chromatin was sheared using a Bioruptor (Diagenode). The lysates were immunoprecipitated (IP) overnight with 4ug anti-RARα ChIP-seq grade antibody (Diagenode: Catalog # C15310155; Lot # A704-001) or control IgG (Diagenode: Catalog # C15410206; Lot # RIG001AK). The immunoprecipitates were then washed, reverse-crosslinked, and subjected to proteinase K digestion. DNA from IP samples and input were eluted using Zymo-spin ChIP kit and quantified using SYBR green qPCR. Forward Primer 5′-TCCTCATTGCCAGCTTCAG-3′; Reverse Primer 5′ CGTGATCTTGCGGATCTTCT-3′. Data are shown as % input.

### Downstream power analysis of PPR

An adequate number of samples is required in order to control the false positive (type I error) and false negative rates (type II error) when conducting hypothesis testing. Low-powered studies often produce more false negatives compared to high-powered studies. As part of our strategy to identify key cluster-specific TFs based on PPR, we developed a strategy based on hypothesis testing with control of the type I error, allowing for sufficient statistical power when identifying significant elements between two distributions.

For a given TF, the correlation of its regulatees is considered by performing principal components analysis (PCA) using the PageRank *z*-scores of its regulatees, thus obtaining a set of principal components that account for 90% of the data variance. The Hotelling T-Squared test, which has previously been used to compact the small sample size issues in microarray analysis, was applied to the regulatee dimension reduction matrix for each TF. Let ${\chi }_{{ik}}=[{\chi }_{{ik}1},{\chi }_{{ik}2},\ldots,{\chi }_{{ikp}}]{\prime}$ be the data for patient *p* in group *i* (RA cluster), with *k* measurement variables (principal components). Here, let *n*_1_ be the number of patients in group 1, *n*_2_ be the number of patients in group 2, *μ*_1*k*_ denote the mean value for the distribution of measurement variable *k* in group 1, and *μ*_2*k*_ denote the mean value for the distribution of measurement variable *k* for group 2. The Hotelling’s T-squared test is based on the assumption that the covariance matrices for measurement variables in the two underlying groups are equal, so under the null hypothesis, we can calculate the assumed pooled common covariance matrix. Thus, the Hotelling’s T-squared test can be conducted as follows (Eq. 3):

*H*_0_: *all the μ*_1*k*_ = *μ*_2*k*_

*H*_1_: *at least one μ*_1*k*_ ≠ *μ*_2*k*_

3 $$F=\frac{{n}_{1}+{n}_{2}-k-1}{k({n}_{1}+{n}_{2}-2)}{T}^{2} \sim {F}_{k,{n}_{1}-k-1}\,{{{{{\rm{where}}}}}}\,{T}^{2}=(\overline{{\chi }_{1}}-\overline{{\chi }_{2}})^{\prime} {\left\{{S}_{p}\left(\frac{1}{{n}_{1}}+\frac{1}{{n}_{2}}\right)\right\}}^{-1}(\overline{{\chi }_{1}}-\overline{{\chi }_{2}})$$

This strategy takes into account any potential relationships between regulatees and also gains increased statistical power because it uses a larger set of regulatees for each hypothesis test in identifying significant TFs.

### Histone Chip-seq and EpiSig

Histone ChIP-seq data for six core histone modifications (H3K4me1, H3K4me3, H3K9me3, H3K27ac, H3K27me3 and H3K36me3) were downloaded from ref. 8. EpiSig, an unsupervised learning approach to simultaneously cluster and align sequencing patterns without prior knowledge^8^, was re-run solely for the RA FLS lines in this study. 78,598 signal-enriched 5 kbp regions were clustered into 245 co-modified 5kbp clusters (Supplementary Figs. 4 and 7). Cluster analysis was performed by variability assessment and significance testing. Variable regions were then mapped to genes using the GREAT analysis tool^40^.

### siRNA transfection

6 Primary RA FLS cell lines (highest ranked by RARα PPR: RA6, RA1, RA4 (CL1) and lowest ranked by RARα PPR: RA10, RA11, RA9 (CL2)) were cultured (at 5% CO2, 37 °C) in Dulbecco’s modified Eagle’s medium (DMEM) supplemented with L-glutamine, gentamicin, penicillin/streptomycin, and 10% heat-inactivated fetal bovine serum (FBS) as previously described^41^. Optimal RARα knockdown by siRNA transfection was performed as previously described^42^ and assessed in time course experiments. Cells were transfected with ON-TARGETplus SMARTpool siRNA targeting human *RARα* (Dharmacon) or ON-TARGET plus Nontargeting Pool siRNA (Dharmacon) using the Human Dermal Fibroblast Nucleofector Kit (Lonza) and cultured in 10%FCS for 3 days. These pools include multiple targeting or matched control siRNA sequences. Cells were serum starved for 18 h in 0.1%FCS/DMEM and subsequently treated with IL-1 (2 ng/ml) for 6 h. RNA was extracted using the RNeasy kit (Qiagen) and qPCR was performed using a cell-based standard approach^43^. Ct (threshold cycle) values were normalized to glyceraldehyde phosphate dehydrogenase (GAPDH).

### Functional assays of gene expression

FLS were stimulated with IL-1b for 6 h, after which RNA was extracted and processed for qPCR. Ct values were then normalized to glyceraldehyde 3-phosphate dehydrogenase (GAPDH).

### Western blot analysis

After transfection, RA FLS were cultured for 3 days. For CDKN2B analysis, cells were serum-starved for 18 h in 1%FCS/DMEM, and subsequently treated with IL-1 (2 ng/ml) for 24 h. FLS were lysed with RIPA buffer (Invitrogen) plus Protease Inhibitor Cocktail (Sigma). The protein concentrations in the lysate extracts were determined with the DC protein assay kit (Bio-Rad). Protein samples from FLS lysates (25–30 μg) were loaded into a NuPAGE 4–12% Bis Tris gel (Invitrogen) and transferred to a Polyvinylidene difluoride (PVDF) membrane (ThermoFisher). After blocking with Tris-buffered saline plus 0.1% Tween 20 (TBST) containing 5% non-fat milk for 1 h at room temperature, the membranes were incubated with mouse monoclonal RARα antibody (Santa Cruz: Catalog# sc-515796, Lot #B0421) and rabbit monoclonal CDKN2B antibody (Cell Signaling Technology: Catalog# 36303, lot#1) diluted 1:1000, and GAPDH antibody diluted 1:5000 (Cell Signaling Technology) at 4 °C overnight. The membranes were washed three times and incubated with horseradish peroxidase-conjugated anti-rabbit and anti-mouse IgG antibodies for 1 and 2 h, respectively. The protein visualization was performed with Clarity Western ECL substrate (Bio-Rad) and the quantitation was performed using Versadoc Imaging system and Quantity One software (Bio-Rad) version 4.6.6.

### Proliferation assay

11 RA FLS were cultured in triplicate in DMEM containing 10% FBS. Cell proliferation was assayed after 4 h incubation with MTT (3-(4,5-dimethylthiazol-2-yl)−2,5-diphenyl tetrazolium bromide) at 1 and 5 days after plating^44^. Fluorescence was quantified using a plate reader with Tecan i-Control 2.0 software.

### Invasion assay

6 Primary RA FLS cell lines (highest ranked (CL1) and lowest ranked (CL2) by RARα PPR) were plated on a Matrigel-coated invasion chamber (Corning). After 4 h serum starvation, the medium was replaced, and TGFβ (20 ng/ml) was added to the top chamber. After 24 h incubation, chamber inserts were fixed in 100% methanol and stained with crystal violet solution. The migrated cells on the bottom of the membrane were quantified using ImageJ software. Blinded quantification was done either manually or in a semi-automatic manner via adjustment thresholds, binarising images and subsequently using the ‘Analyze particle’ tool. Fold changes for TGFβ-induced invasion were calculated by normalizing on respective controls with media alone.

### Phospho SMAD2/3 ELISA

8 primary RAFLS were plated, and serum starved for 18 h in 0.1%FCS/DMEM and subsequently treated with TGFβ (20 ng/ml) for 30 min. Cell lysates were collected and phospho SMAD2/3 and total SMAD2/3 (Cell Signaling Technology) were measure by ELISA, according to the manufacturer’s protocol. Fluorescence was quantified using a plate reader with Tecan i-Control 2.0 software.

### TGFβ ELISA

After transfection, 8 primary RAFLSs were seeded in 24-well plates and cultured in DMEM with 10% FCS at 37 °C for 24 h. Cells were serum starved for 18 h in 1%FCS/DMEM and then treated with IL-1 (2 ng/ml) for 24 h. TGFβ in cell culture supernatants were measure by ELISA (R&D system) according to the protocols provided by the manufacturer. Fluorescence was quantified using a plate reader with Tecan i-Control 2.0 software.

### All-trans retinoic acid treatment and proliferation assay

CL1 and CL2 FLS were plated in triplicate in a 96-well plate (2 × 103 cells/well) with DMEM containing 10% FBS. After 24 h, medium was replaced by a serum-deprived (1% FBS) medium containing 1 μM of ATRA (Sigma-Aldrich, St. Louis, MO, USA) or with DMSO for 1 h. PDGF-BB (10 ng/ml) was added to the wells and cells were incubated for 5 and 7 days. Cell growth was determined after 4 h incubation with MTT (3-(4,5-dimethylthiazol-2-yl)−2,5-diphenyl tetrazolium bromide) and was read at 550 nm with a spectrophotometer. Fold change in cell growth was calculated by normalizing each cell line with its median value from day 1.

### Reporting summary

Further information on research design is available in the Nature Research Reporting Summary linked to this article.

## Supplementary information

Supplementary Information

Description of Additional Supplementary Files

Supplementary Dataset 1

Supplementary Dataset 2

Supplementary Dataset 3

Reporting Summary

## Data Availability

The ATAC-seq, RNA-seq, and ChIP-seq data that support the findings of this study have been previously deposited in the Gene Expression Omnibus with the primary accession code GSE112658 and in the Database of Genotypes and Phenotypes (dbGaP) with the dbGaP study accession code phs001615.v1.p1. The EpiTensor promoter-enhancer interaction data and CIS-BP motif input reference data used, along with the PageRank and expression data generated in this study, have been deposited in the Figshare collection [10.6084/m9.figshare.c.6152160]. Source data are provided with this paper.

## Source data

Source Data

## Code availability

The Taiji multi-omics bioinformatics pipeline used to analyze the data is available at: https://github.com/Taiji-pipeline/Taiji
