# Supplementary material for: Systems-biology analysis of rheumatoid arthritis fibroblast-like synoviocytes implicates cell line-specific transcription factor function
Source: Nat Commun. 2022 Oct 20;13:6221. doi: 10.1038/s41467-022-33785-w (PMC9584907; doi:10.1038/s41467-022-33785-w)
Supplement: Supplementary file 1 — Supplementary Information [file 41467_2022_33785_MOESM1_ESM.pdf]

Systems-biology analysis of rheumatoid arthritis fibroblast-like synoviocytes reveals cell line-specific transcription factor function

Supplementary Discussion

E2F7/E2F1 antagonistic CL2 signature

The transcriptional repressor E2F7 is the rank 3 cluster-specific TF (Fig. 1c) (q-value = 7.1×10^{-3}). This prediction is driven by it's differential expression (3.89-fold CL2, p-value = 4.2×10^{-4}) (a factor in determining PPR via edge weight construction: see Methods) since the other important metric governing PPR, network connectivity, is relatively low for E2F7, as illustrated by its peripheral position and low out-degree in the CL2 TF-TF subnetwork (Fig. 3b). The transcriptional activator E2F1 (2.83-fold CL2, p-value = 5.4×10^{-3}), and E2F7 are mutually antagonistic and regulate proliferation, differentiation, apoptosis and responses to DNA-damage.[1] However, we observe that they are both up-regulated in CL2. Paradoxically, some cancers, such as head and neck squamous cell carcinomas (HNSCCs) also exhibit the overexpression of both TFs. Mislocalization of E2F7 from the nucleus to the cytoplasm via XPO1 nuclear export is a feature in 80% of HNSCCs leading to aberrant differentiation, increased proliferation and drug resistance.[2] In our data, we observe a strong correlation between E2F7 and XPO1 transcript levels (Pearson R = 0.92) indicating that, despite increased transcription of E2F7 in CL2, there is also increased XPO1 available to potentially export the E2F7 protein from the nucleus. Indeed, known transcriptional targets of E2F7 repression such as Rac GTPase activating protein 1 (RACGAP1) (2.26-fold CL2, p-value = 1.4×10^{-2}) and DNA-damage response genes such as RAD51[3] (2.51-fold CL2, p-value = 6.8×10^{-3}) are significantly more highly expressed in CL2 despite the approximately 4-fold higher mRNA levels of E2F7 in CL2 compared to CL1. We regard these findings as observational and note that many other mechanisms may also “inactivate” E2F7 transcriptional effects such as post-translational modifications and mutations in the sequence of the E2F7 protein.

Supplementary References

1. Dimova, D. K. & Dyson, N. J. The E2F transcriptional network: old acquaintances with new faces. *Oncogene* **24**, 2810–2826 (2005).
2. Saenz-Ponce, N. *et al.* Targeting the XPO1-dependent nuclear export of E2F7 reverses anthracycline resistance in head and neck squamous cell carcinomas. *Sci. Transl. Med.* **10**, (2018).
3. Mitxelena, J. *et al.* An E2F7-dependent transcriptional program modulates DNA damage repair and genomic stability. *Nucleic Acids Res.* **46**, 4546–4559 (2018).

Supplementary Figures

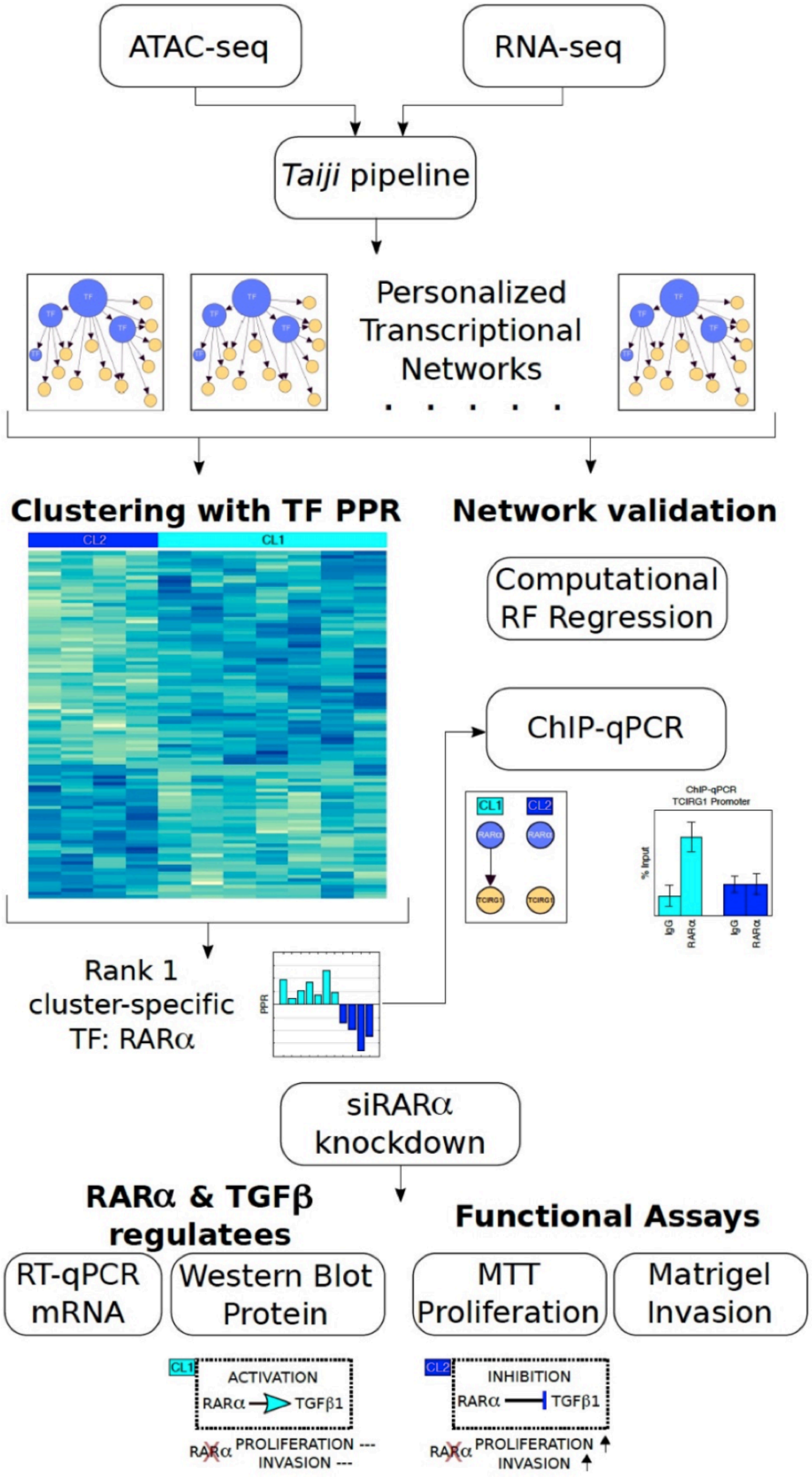

Supplementary Figure 1

Computational and experimental pipeline overview.

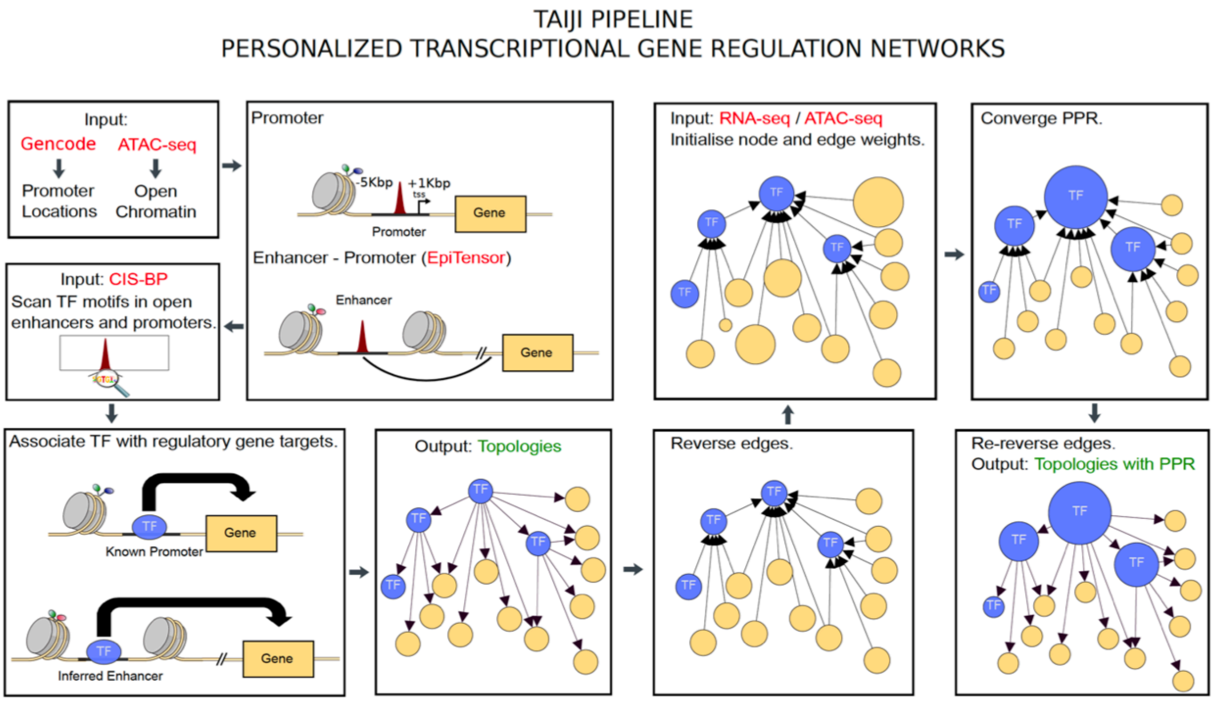

Supplementary Figure 2

Extended representation of the Taiji Integrative Pipeline for construction of patient-specific global transcriptional gene regulation networks.

a

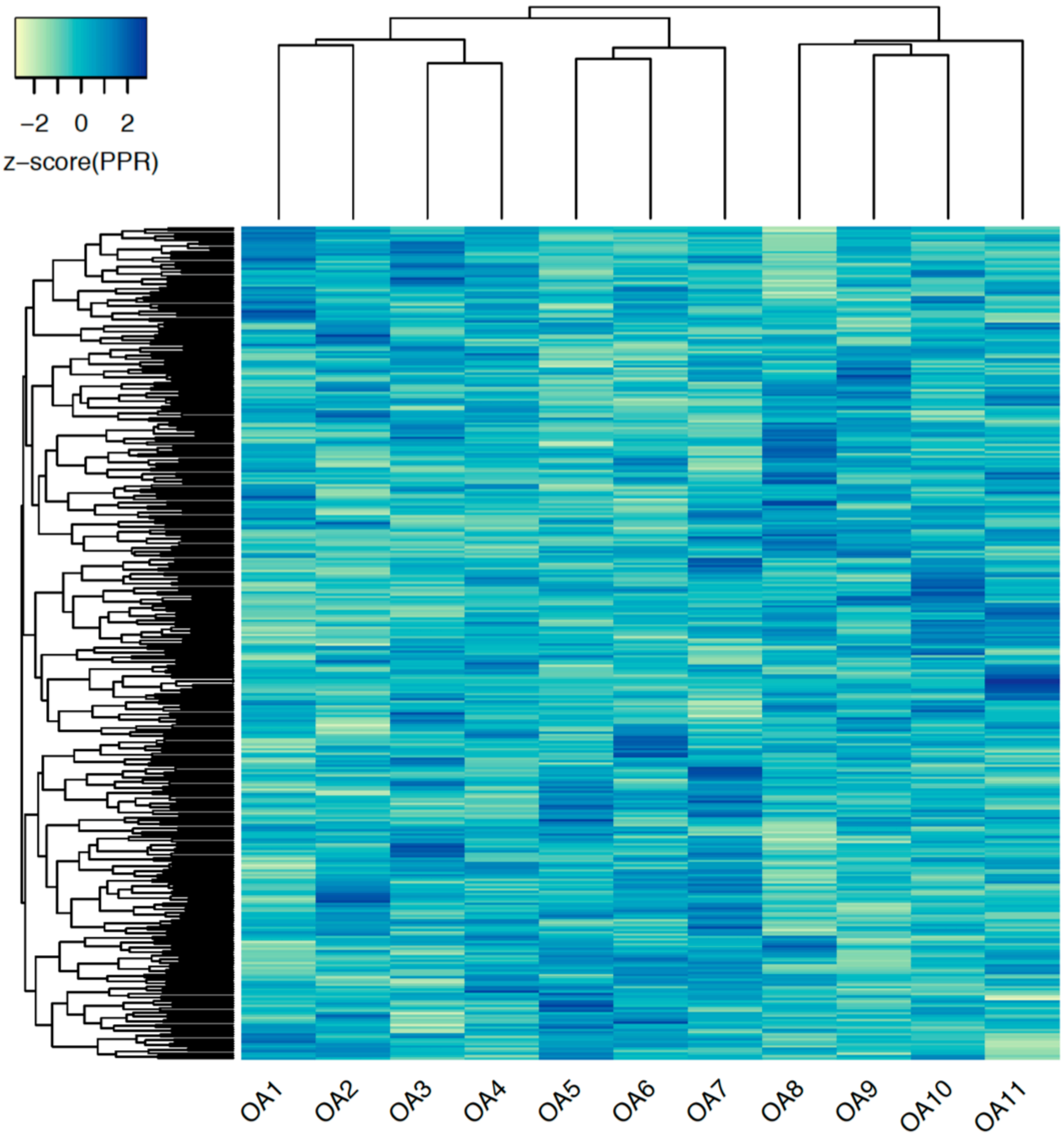

.

b

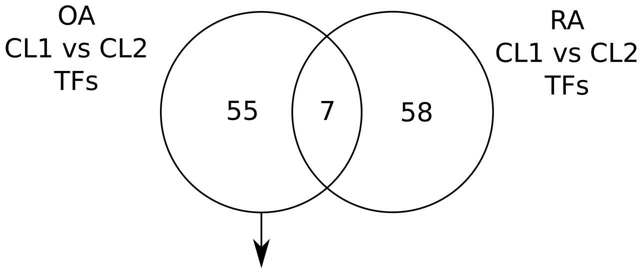

Pathway name	Entities found	Entities Total	Entities ratio	Entities pValue	Entities FDR	Reactions found	Reactions total	Reactions ratio	Species name
Nuclear Receptor transcription pathway	10	86	0.006	2.43E-10	2.69E-8	*2*	*2*	*0*	Homo sapiens
Activation of anterior HOX genes in hindbrain development during early embryogenesis	11	116	0.008	2.47E-10	2.69E-8	*21*	*43*	*0.003*	Homo sapiens
Activation of HOX genes during differentiation	11	116	0.008	2.47E-10	2.69E-8	*21*	*43*	*0.003*	Homo sapiens
Transcriptional regulation of pluripotent stem cells	7	45	0.003	1.89E-8	1.55E-6	*11*	*35*	*0.003*	Homo sapiens
Interleukin-4 and Interleukin-13 signaling	11	211	0.015	1.1E-7	7.12E-6	*19*	*47*	*0.003*	Homo sapiens
Generic Transcription Pathway	26	1,555	0.107	2.6E-6	1.41E-4	*71*	*824*	*0.061*	Homo sapiens
STAT3 nuclear events downstream of ALK signaling	4	18	0.001	6.01E-6	2.77E-4	*10*	*10*	*0.001*	Homo sapiens
RNA Polymerase II Transcription	26	1,694	0.116	1.23E-5	5.06E-4	*71*	*885*	*0.065*	Homo sapiens
Nuclear events stimulated by ALK signaling in cancer	4	27	0.002	3E-5	1.05E-3	*4*	*9*	*0.001*	Homo sapiens
PTK6 Expression	3	10	0.001	4E-5	1.25E-3	*3*	*3*	*0*	Homo sapiens
Gene expression (Transcription)	26	1,855	0.128	6E-5	1.73E-3	*73*	*1,000*	*0.074*	Homo sapiens
FOXO-mediated transcription	6	110	0.008	7.66E-5	2.07E-3	*13*	*85*	*0.006*	Homo sapiens
Regulation of gene expression by Hypoxia-inducible Factor	3	15	0.001	1.29E-4	3.22E-3	*7*	*7*	*0.001*	Homo sapiens
Signaling by ALK	4	43	0.003	1.74E-4	3.99E-3	*13*	*40*	*0.003*	Homo sapiens
Interferon alpha/beta signaling	7	186	0.013	1.9E-4	3.99E-3	*18*	*22*	*0.002*	Homo sapiens
Signaling by Interleukins	13	643	0.044	2.08E-4	4.15E-3	*102*	*493*	*0.036*	Homo sapiens
Signaling by cytosolic FGFR1 fusion mutants	3	23	0.002	4.47E-4	7.27E-3	*8*	*14*	*0.001*	Homo sapiens
FOXO-mediated transcription of cell death genes	3	23	0.002	4.47E-4	7.27E-3	*5*	*15*	*0.001*	Homo sapiens
Cytokine Signaling in Immune system	17	1,092	0.075	4.62E-4	7.27E-3	*141*	*708*	*0.052*	Homo sapiens
NGF-stimulated transcription	4	56	0.004	4.7E-4	7.27E-3	*6*	*37*	*0.003*	Homo sapiens

Supplementary Figure 3

(**a**) Hierarchical clustering of 11 OA cell lines using the top 350 TFs ranked by variance in their PPR leads to two clusters containing 7 OA patients and 4 OA patients, OA clusters 1 (OA CL1) and 2 (OA CL2) respectively, at the first split in the dendogram. 62 TFs were found to have significantly different (p-value <0.05) PPR values from a two-tailed Students t-test between OA CL1 and OA CL2. Only 7 of which were common to the 65 differential TFs between RA CL1 and CL2 illustrating disease specificity. (**b**) Functional enrichment analysis of the 62 OA cluster-specific TFs results in developmental pathways including: *Activation of HoX genes during differentiation* (p-value = 2.47×10^{-10} from hypergeometric test).

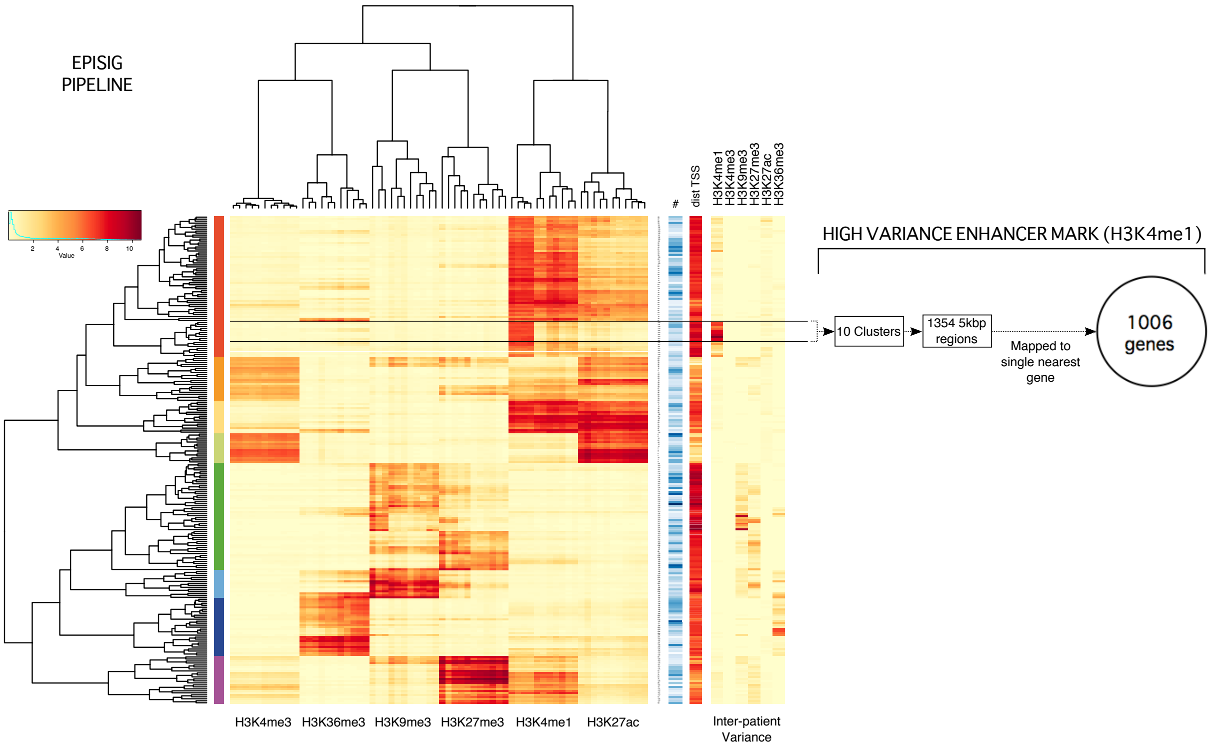

GO BIOLOGICAL PROCESS

Term Name	Binom Rank	Binom Raw P-Value	Binom FDR Q-Val	Binom Fold Enrichment	Binom Observed Region Hits	Binom Region Set Coverage	Hyper Rank	Hyper FDR Q-Val	Hyper Fold Enrichment	Hyper Observed Gene Hits	Hyper Total Genes	Hyper Gene Set Coverage
heart development	2	4.3370e-14	2.8505e-10	2.1279	118	8.71%	4	6.7734e-17	3.1654	80	466	7.95%
cardiac chamber development	10	4.0538e-11	5.3287e-8	2.7166	57	4.21%	22	1.6118e-13	4.6695	39	154	3.88%
cardiac septum development	12	9.8450e-11	1.0784e-7	3.0363	46	3.40%	21	1.1339e-13	5.9541	31	96	3.08%
mesenchyme development	15	1.9538e-10	1.7122e-7	2.4014	66	4.87%	44	6.0437e-10	3.6877	38	190	3.78%
heart morphogenesis	16	3.3796e-10	2.7765e-7	2.3504	67	4.95%	25	1.7495e-12	3.7865	46	224	4.57%
cardiac septum morphogenesis	17	3.8320e-10	2.9630e-7	3.3179	38	2.81%	24	7.5621e-13	7.0917	25	65	2.49%
cardiac chamber morphogenesis	20	5.9494e-10	3.9102e-7	2.7906	48	3.55%	28	1.2589e-11	4.9169	32	120	3.18%
mesenchymal cell differentiation	24	8.8620e-10	4.8538e-7	2.6274	52	3.84%	80	4.4421e-8	3.9713	28	130	2.78%
regulation of organ morphogenesis	29	1.6859e-9	7.6417e-7	2.3259	63	4.65%	90	9.1175e-8	2.9620	40	249	3.98%
regulation of canonical Wnt signaling pathway	33	5.7069e-9	2.2732e-6	2.4323	54	3.99%	185	2.1782e-5	2.6013	34	241	3.38%
palate development	35	6.9612e-9	2.6144e-6	2.9748	38	2.81%	159	6.6862e-6	4.0083	20	92	1.99%
extracellular matrix organization	36	7.0844e-9	2.5868e-6	2.3293	58	4.28%	222	8.3209e-5	2.3048	38	304	3.78%
extracellular structure organization	37	7.4443e-9	2.6447e-6	2.3260	58	4.28%	226	8.8615e-5	2.2972	38	305	3.78%
regulation of epithelial cell proliferation	39	9.3714e-9	3.1586e-6	2.1156	70	5.17%	78	2.8893e-8	2.8611	45	290	4.47%
outflow tract morphogenesis	47	2.6420e-8	7.3891e-6	2.9774	35	2.58%	49	1.1234e-9	5.9654	22	68	2.19%
regulation of Wnt signaling pathway	48	3.2416e-8	8.8773e-6	2.1001	66	4.87%	196	3.9189e-5	2.3266	40	317	3.98%
regulation of morphogenesis of an epithelium	49	5.6607e-8	1.5186e-5	2.4864	45	3.32%	188	2.5121e-5	2.9004	28	178	2.78%
regulation of epithelial cell migration	51	5.9997e-8	1.5464e-5	2.3027	52	3.84%	71	1.2207e-8	3.5073	35	184	3.48%
positive regulation of epithelial cell proliferation	67	1.7691e-7	3.4709e-5	2.4135	44	3.25%	208	5.6937e-5	2.9054	26	165	2.58%
positive regulation of epithelial cell migration	68	1.8415e-7	3.5597e-5	2.6110	38	2.81%	115	1.1214e-6	3.8480	24	115	2.39%

ABI3BP ADAMTS20 ADAMTS5 CCDC80 COL11A1 COL1A2 COL3A1 COL5A2 COL8A1 DCN DPT FBLN5 FBN1 FLRT2 HAS2 ITGA1 ITGA4 ITGB1 ITGB6 LAMA2 LAMA3 LAMA4 LAMC1 MATN1 MMP16 P4HA1 POSTN RECK SFRP2 SOX9 SPOCK2 TGFB2 THBS1 TLL1 TNC TNFRSF11B VCAM1 VCAN

BMP4 BMP5 CDH13 CDK6 DLG1 DLX6 DUSP10 FBXW7 FGF7 FRS2 HAS2 HMGB2 HTR2B IFT57 IGF1 IL6 KLF9 LAMC1 MEF2C MTSS1 NFIB NOG NR2F2 NRP1 NRP2 PRKCA PRKD1 PTN PTPRM RGCC ROBO1 RTN4 SFRP2 SNAI2 SOX2 SOX9 TACR1 TGFB2 THBS1 TNFAIP3 TP63 TWIST1 VEGFC WNT2 WNT5A

GO MOLECULAR FUNCTION

Term Name	Binom Rank	Binom Raw P-Value	Binom FDR Q-Val	Binom Fold Enrichment	Binom Observed Region Hits	Binom Region Set Coverage	Hyper Rank	Hyper FDR Q-Val	Hyper Fold Enrichment	Hyper Observed Gene Hits	Hyper Total Genes	Hyper Gene Set Coverage
SMAD binding	3	1.7895e-8	2.5166e-5	3.3761	30	2.22%	7	5.0301e-3	3.7375	15	74	1.49%
bHLH transcription factor binding	13	9.3727e-5	3.0418e-2	3.6240	13	0.96%	15	3.1638e-2	5.0864	8	29	0.80%
E-box binding	15	1.1757e-4	3.3070e-2	3.3448	14	1.03%	17	3.8058e-2	4.3670	9	38	0.89%

ACVR1 AXIN2 BMPR1B COL1A2 COL3A1 COL5A2 DAB2 DROSHA HMGA2 RGCC SMAD7 TCF12 TGFBR2 ZEB2

Supplementary Figure 4

Heatmap hierarchically clustering 245 EpiSig co-modified clusters derived from 78,598 signal-enriched 5kbp regions based on Histone ChIP-seq data for six core histone modifications

(H3K4me1, H3K4me3, H3K9me3, H3K27ac, H3K27me3 and H3K36me3). Each column represents a single RA patient FLS cell line for a specific mark. Rows cluster into putative regulatory regions (e.g. pale green is active promoter marked by H3K4me3 and H3K27ac). Columns cluster by mark. Reading left to right, main heatmap is followed by columns: cluster id, number of 5kbp regions, distance to TSS and inter-patient variance for each mark. 10 high variance enhancer clusters marked, which map to 1,006 genes. Tables for the top significantly enriched (using Mann-Whitney test) GO Biological Process' and GO Molecular Function categories for the 1,006 genes.

a.

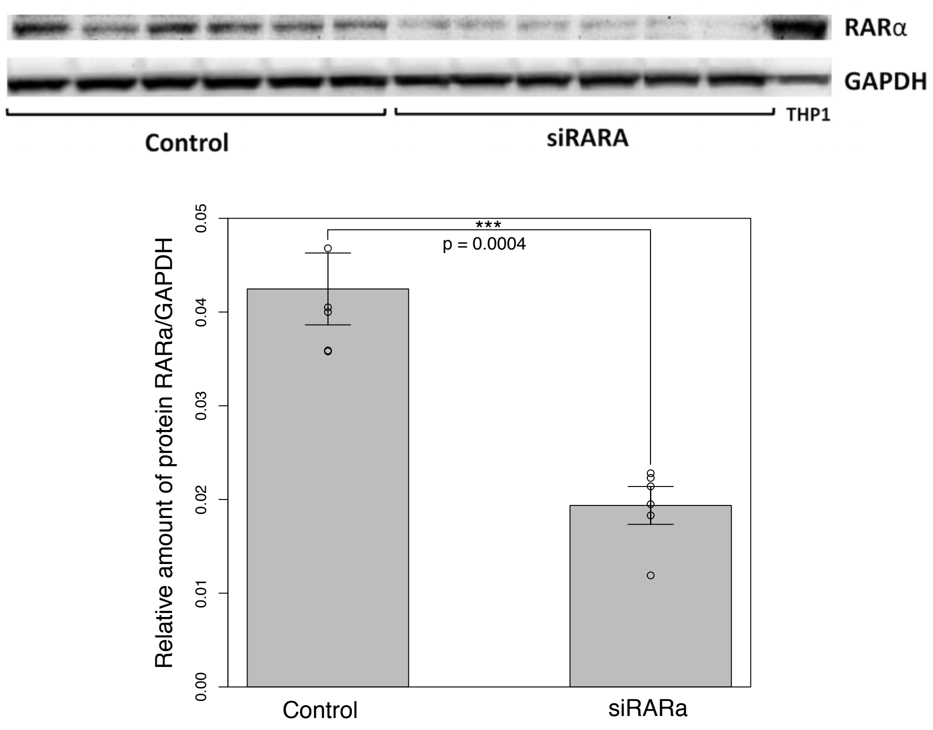
RARα
GAPDH
Control
siRARA
THP1
Relative amount of protein RARa/GAPDH
p = 0.0004
Control
siRARa

b.

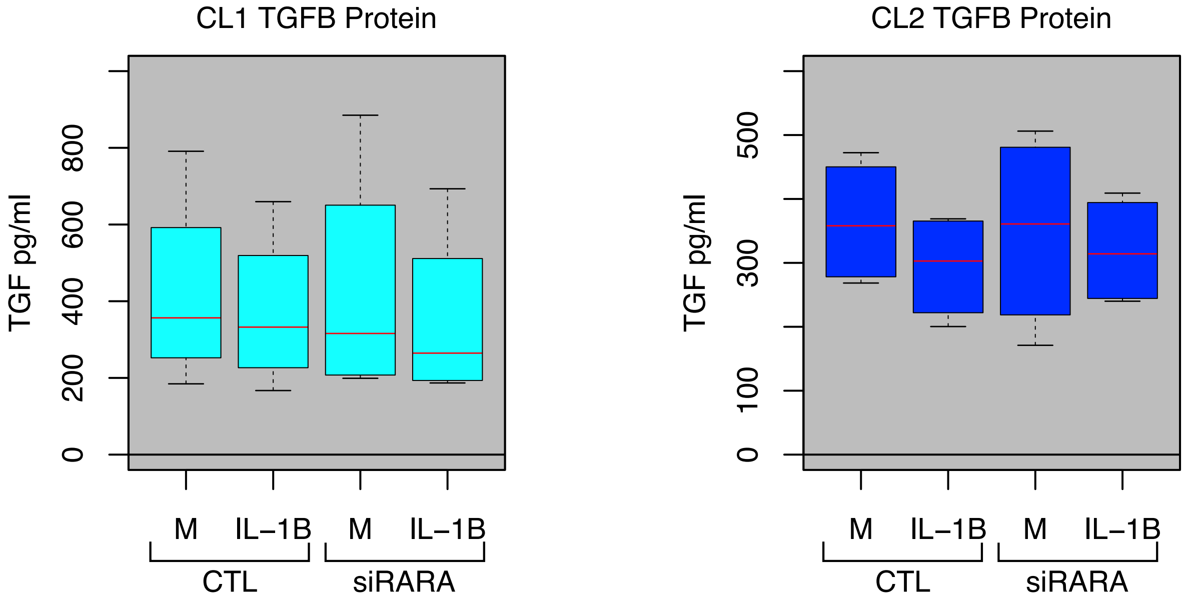
CL1 TGFB Protein
TGF pg/ml
M
IL−1B
M
IL−1B
CTL
siRARA
CL2 TGFB Protein
TGF pg/ml
M
IL−1B
M
IL−1B
CTL
siRARA

c.

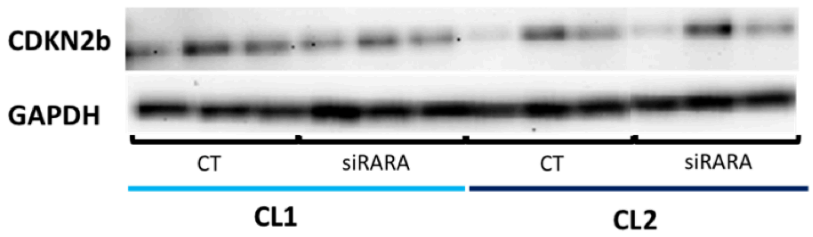

d.

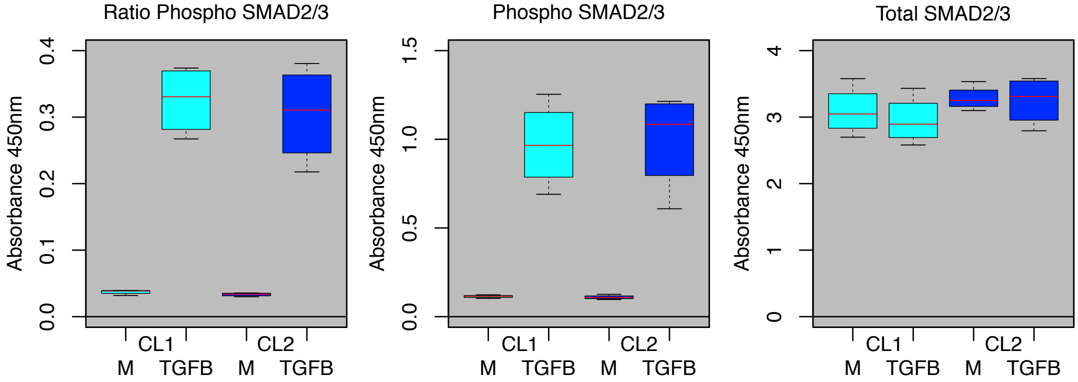

Supplementary Figure 5

a. Western blot analysis of RARα knockdown efficiency in RA-FLS lines. 6 biologically independent RA-FLS cell lines were transfected with 1ug of RARα siRNA (Human RARα siRNA smartpool, Dharmacon) and control (Non-targeting Control Pool, Dharmacon) and plated for 3 days. The RARα protein expression was analyzed by Western blot, using 1:1000 dilution of RARα mouse antibody (Santa Cruz) and normalized to GAPDH (Cell Signaling). The protein level indicates 53% to 58% of RARα inhibition ($p=0.004$). Barplot centre line is mean and error bars +/- 0.5 s.d. Band intensities were quantified using Versadoc Quantity One 4.6.6, and the statistical significance was determined by two-tailed paired Student's t-test.

b. TGFB protein levels by Elisa in 1%FCS/DMEM and then treated with IL-1 (2ng/ml) for CL1 and CL2 lines with (siRARA) and without (CTL) RARA knockdown. CL1 n=4 and CL2 n=4

biologically independent cell lines. Red centre line for median, whiskers represent maximum and minimum values, box width from quartile 1 to quartile 3.

c. CDKN2B western blot performed once for CL1 and CL2 (CL1 n=3 and CL2 n=3 biologically independent cell lines) treated with IL1 IL-1 (2ng/ml) with (siRARA) and without (CTL) RARA knockdown.

d. Phospho SMAD2/3 by Elisa with and without TGFB treatment for CL1 and CL2 lines. CL1 n=4 and CL2 n=4 biologically independent cell lines. Red centre line for median, whiskers represent maximum and minimum values, box width from quartile 1 to quartile 3.

Source data are provided as a Source Data file.

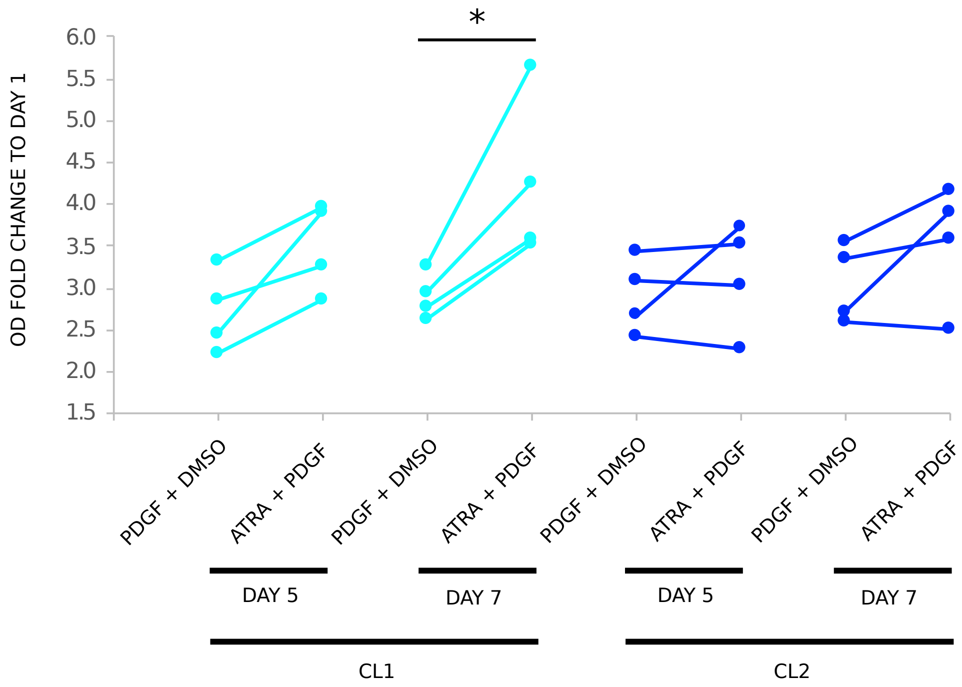

Supplementary Figure 6

The RARA ligand ATRA has differential effects on CL1 and CL2 FLS growth. CL1 at Day 7 exhibited increase in proliferation under ATRA (p=0.0315 by two-tailed paired Student's t-test). FLS were cultured with 1 uM of ATRA in either medium or 10 ng/ml of PDGF. Cell growth was quantified using an MTT assay. *: p<0.05

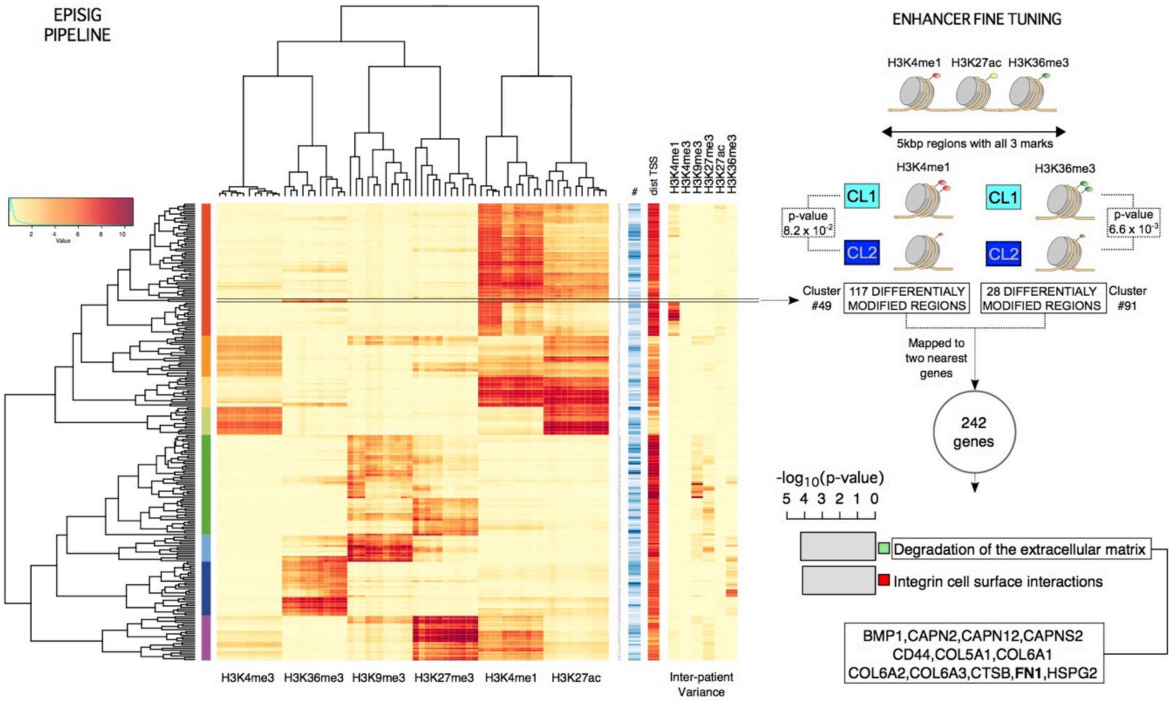

Supplementary Figure 7

Heatmap as per Figure S4 legend. 2 enhancer clusters with significant CL1 vs CL2 difference in EpiSig signal marked, which map to 242 genes. Functional enrichment analysis of the 242 genes.

Supplementary Tables

Taiji prediction	Experimental validation
Molecular events	
RARa binding CL1 > CL2 at RARa binding motif	Confirmed with ChIP PCR at TCIRG1 promoter
RARa deficiency: differential effect on CL1 and CL2 TGFß mRNA levels	Confirmed for TGFß by RT-qPCR
RARa deficiency: differential effect on CL1 and CL2 TGFß regulatees	Confirmed for CDK2NB, ROCK1, CCND1 expression by RT-qPCR.
RARa deficiency: differential effect on CL1 and CL2 TGFß regulatees	Confirmed for CDK2NB protein by Western Blot
Normal TGFß signaling in CL1 and CL2	Confirmed using P-SMAD assay after stimulating cells with TGFß. Showed that explanation for decreased TGFß regulatee was not due to deficient signaling
RARa deficiency: differential effects on EMT markers in CL1 and CL2	Confirmed based on FN1 and VIM expression by RT-qPCR
FLS functions	
RARa deficiency: differential effect on cell invasion in CL1 and CL2	Confirmed in Matrigel assay
RARa deficiency: differential effect on cell proliferation in CL1 and CL2	Confirmed by MTT assay
CL1 and CL2 cells phenotypically different.	Confirmed by cell size analysis using flow cytometry and image analysis (data not shown)
Proliferation of CL1< CL2	Confirmed by cell counting in culture
RARa agonist (ATRA): differential effects on CL1 and CL2 proliferation	Confirmed by MTT assay

Supplementary Table 1

Summary of computational predictions and experimental validation.

Marker	%	stdev
CD34+	2.0	0.8
CD90+	95.9	2.4
FAPα+	90.5	4.9
CD14+	2.0	1.1
PDPN+	92.0	2.3
CD90+, FAPα+, PDPN+	82.5	6.7
CD90+, CD14+, PDPN-	1.8	1.0

Supplementary Table 2

Flow data on FLS phenotype.
