# Supplementary material for: Systems-biology analysis of rheumatoid arthritis fibroblast-like synoviocytes implicates cell line-specific transcription factor function
Source: Nat Commun. 2022 Oct 20;13:6221. doi: 10.1038/s41467-022-33785-w (PMC9584907; doi:10.1038/s41467-022-33785-w)
Supplement: Supplementary file 2 — Description of Additional Supplementary Files [file 41467_2022_33785_MOESM2_ESM.pdf]

Supplementary Data 1: Top 100 cluster-specific transcription factors.

Supplementary Data 2: CL1 vs CL2 differentially expressed genes.

Supplementary Data 3: Enriched pathways for CL1 vs CL2 differentially expressed genes.
