# Supplementary material for: Systems-biology analysis of rheumatoid arthritis fibroblast-like synoviocytes implicates cell line-specific transcription factor function
Source: Nat Commun. 2022 Oct 20;13:6221. doi: 10.1038/s41467-022-33785-w (PMC9584907; doi:10.1038/s41467-022-33785-w)
Supplement: Supplementary file 6 — Reporting Summary [file 41467_2022_33785_MOESM6_ESM.pdf]

nature research

Corresponding author(s): Prof. Wei Wang

Last updated by author(s): Aug 17, 2022

Reporting Summary

Nature Research wishes to improve the reproducibility of the work that we publish. This form provides structure for consistency and transparency in reporting. For further information on Nature Research policies, see our Editorial Policies and the Editorial Policy Checklist.

Statistics

For all statistical analyses, confirm that the following items are present in the figure legend, table legend, main text, or Methods section.

n/a	Confirmed	
☐	☒	The exact sample size (n) for each experimental group/condition, given as a discrete number and unit of measurement
☐	☒	A statement on whether measurements were taken from distinct samples or whether the same sample was measured repeatedly
☐	☒	The statistical test(s) used AND whether they are one- or two-sided<br>*Only common tests should be described solely by name; describe more complex techniques in the Methods section.*
☐	☒	A description of all covariates tested
☐	☒	A description of any assumptions or corrections, such as tests of normality and adjustment for multiple comparisons
☐	☒	A full description of the statistical parameters including central tendency (e.g. means) or other basic estimates (e.g. regression coefficient) AND variation (e.g. standard deviation) or associated estimates of uncertainty (e.g. confidence intervals)
☐	☒	For null hypothesis testing, the test statistic (e.g. F, t, r) with confidence intervals, effect sizes, degrees of freedom and P value noted<br>*Give P values as exact values whenever suitable.*
☒	☐	For Bayesian analysis, information on the choice of priors and Markov chain Monte Carlo settings
☐	☒	For hierarchical and complex designs, identification of the appropriate level for tests and full reporting of outcomes
☐	☒	Estimates of effect sizes (e.g. Cohen's d, Pearson's r), indicating how they were calculated

Our web collection on statistics for biologists contains articles on many of the points above.

Software and code

Policy information about availability of computer code

Data collection	Western blot quantitation was performed using Versadoc Imaging system and Biorad Quantity One software version 4.6.6<br>For the MTT proliferation and Elisa assays, fluorescence was quantified using a plate reader with Tecan i-Control 2.0 software.
Data analysis	Invasion Assay: Image analysis performed with ImageJ version 2.1.0 software. Blinded quantification was done either manually or in a semi-automatic manner via adjustment thresholds, binarising images and subsequently using the 'Analyze particle' tool.<br>Taiji multi-omics bioinformatics pipeline used to analyse data. Code available at: https://github.com/Taiji-pipeline/Taiji<br>EpiSig bioinformatics pipeline used to analyse data. Code available at: http://wanglab.ucsd.edu/star/EpiSig/

For manuscripts utilizing custom algorithms or software that are central to the research but not yet described in published literature, software must be made available to editors and reviewers. We strongly encourage code deposition in a community repository (e.g. GitHub). See the Nature Research guidelines for submitting code & software for further information.

Data

Policy information about availability of data

All manuscripts must include a data availability statement. This statement should provide the following information, where applicable:

- Accession codes, unique identifiers, or web links for publicly available datasets
- A list of figures that have associated raw data
- A description of any restrictions on data availability

The ATAC-seq, RNA-seq and ChIP-seq data that support the findings of this study have been previously deposited in the Gene Expression Omnibus with the primary accession code GSE112658 and in the Database of Genotypes and Phenotypes (dbGaP) with the dbGaP study accession code phs001615.v1.p1.
The EpiTensor promoter-enhancer interaction data and CIS-BP motif input reference data used, along with the PageRank and expression data generated in this study, have been deposited in the Figshare collection [https://doi.org/10.6084/m9.figshare.c.6152160].

Field-specific reporting

Please select the one below that is the best fit for your research. If you are not sure, read the appropriate sections before making your selection.

- [x] Life sciences
- [ ] Behavioural & social sciences
- [ ] Ecological, evolutionary & environmental sciences

For a reference copy of the document with all sections, see nature.com/documents/nr-reporting-summary-flat.pdf

Life sciences study design

All studies must disclose on these points even when the disclosure is negative.

Sample size	The sample size was determined by the number of samples with full data sets previously reported ()Nat Commun. 2018 May 15;9(1):1921). All 11 RA FLS cell lines for which RNA-seq, ATAC-seq and ChiP-seq data were available were used for pipeline analysis and biologic validation Downstream power analysis of TF regulatees were employed to control false positive and false negative rates.
Data exclusions	No data were excluded from the analyses.
Replication	For biologic validation experiments, at least 3 FLS lines were generally cultured in triplicate and repeated at least once to confirm results.
Randomization	Randomization was was not required for the initial analysis because all samples were evaluated the same way. The patients were hierarchically clustered, using the PageRank of the expressed TFs, into 2 clusters (CL1 and CL2). Based on the sample size and study design, patient classification was governed by transcriptional regulatory differences and hypothesis testing was based on this stratification.
Blinding	Samples were deidentified for pipeline and biological validation studies. The individuals performing the analyses did not have clinical data available.

Reporting for specific materials, systems and methods

We require information from authors about some types of materials, experimental systems and methods used in many studies. Here, indicate whether each material, system or method listed is relevant to your study. If you are not sure if a list item applies to your research, read the appropriate section before selecting a response.

Materials & experimental systems

n/a	Involved in the study	
[ ]	[x]	Antibodies
[ ]	[x]	Eukaryotic cell lines
[x]	[ ]	Palaeontology and archaeology
[x]	[ ]	Animals and other organisms
[ ]	[x]	Human research participants
[x]	[ ]	Clinical data
[x]	[ ]	Dual use research of concern

Methods

n/a	Involved in the study	
[x]	[ ]	ChIP-seq
[x]	[ ]	Flow cytometry
[x]	[ ]	MRI-based neuroimaging

Antibodies

Antibodies used	Antibodies purchased from Diagenode for ChIP assay:<br>RARa: Catalog # C15310155; Lot # A704-001<br>IgG: Catalog # C15410206; Lot # RIG001AK<br>Antibodies purchased from Santa Cruz for Western Blot assay:<br>RARA: Catalog# sc-515796, Lot #B0421<br>Antibodies purchased from Cell Signaling Technology for Western Blot assay:<br>CDKN2B: Catalog# 36303, lot#1
Validation	Positive and negative controls were included in each experiment.<br>RARa, Manufacturer Diagenode, Catalog # C15310155 - manufacturer recommendation: 4 µl/ChIP<br>https://www.ncbi.nlm.nih.gov/pmc/articles/PMC4372260/<br>https://www.ncbi.nlm.nih.gov/pmc/articles/PMC6338086/<br>RARa, Manufacturer Santa Cruz, Catalog# sc-515796, manufacturer recommendation: dilution range 1:100-1:1000)<br>https://link.springer.com/article/10.1007/s12272-019-01170-9<br>https://www.ncbi.nlm.nih.gov/pmc/articles/PMC8997856/<br>CDKN2B, Manufacturer Cell Signaling Technology, Catalog# 36303, manufacturer recommendation dilution: 1:1000<br>https://www.ncbi.nlm.nih.gov/pmc/articles/PMC7950249/

Eukaryotic cell lines

Policy information about cell lines

Cell line source(s)	Primary FLS were cultured from synovial tissue obtained from patients with RA at the time of total joint replacement or synovectomy.
Authentication	Cell lines are de-identified at the time of synovial disaggregation and give a code number with a key known only to Dr. Firestein and Professor Boyle. Each aliquot of the frozen cells is labeled with the cell code, and every flask containing the cell line is also labeled with the code.
Mycoplasma contamination	Mycoplasma tests are performed on a regular basis.
Commonly misidentified lines (See ICLAC register)	None

Human research participants

Policy information about studies involving human research participants

Population characteristics	RA patients were female and the mean ages of patients were 55±9. Of the 7 RA patients with clinical information available, treatment included NSAIDs (n=5), low dose prednisone (<5mg/d) (n=4), methotrexate (n=4), methotrexate plus leflunomide (n=1), TNF blocker (n=3), IL-6 blocker (n=1). OA patients were female and the mean ages of patients were 66 ±12. Therapy information was available for 5 OA patients, who were treated with NSAIDs (n=2) or analgesics.
Recruitment	Patients were recruited from orthopedic clinics based on the plan for joint replacement surgery. All were consented prior to surgery for use of remnant samples as per UCSD IRB protocol 14-0175.
Ethics oversight	The protocol for collecting and studying FLS was approved by the UCSD IRB (protocol 14-0175)

Note that full information on the approval of the study protocol must also be provided in the manuscript.
